# Beta1-adrenoceptor antagonist, metoprolol attenuates cardiac myocyte Ca^2+^ handling dysfunction in rats with pulmonary artery hypertension

**DOI:** 10.1016/j.yjmcc.2018.05.015

**Published:** 2018-07

**Authors:** Ewan D. Fowler, Mark J. Drinkhill, Ruth Norman, Eleftheria Pervolaraki, Rachel Stones, Emma Steer, David Benoist, Derek S. Steele, Sarah C. Calaghan, Ed White

**Affiliations:** a Multidisciplinary Cardiovascular Research Centre, University of Leeds, Leeds, UK; b School of Physiology, Pharmacology and Neuroscience, Faculty of Biomedical Sciences, University of Bristol, Bristol, UK; c L'institut de rythmologie et modélisation cardiaque, Inserm U-1045, Université de Bordeaux, Bordeaux, France

**Keywords:** Pulmonary artery hypertension, Right heart failure, Beta-blocker, Cardiac myocyte, Ca^2+^ handling, Monocrotaline, BB, beta blocker, BIN-1, amphiphysin II, JP-2, junctophilin 2, LTCC, L-type Ca^2+^ channel, MCT, monocrotaline, PAH, pulmonary arterial hypertension, PLN, phospholamban, RV, right ventricle, SCW, spontaneous Ca^2+^ wave, SR, sarco(endo)plasmic reticulum, WCE, whole-cell event

## Abstract

Right heart failure is the major cause of death in Pulmonary Artery Hypertension (PAH) patients but is not a current, specific therapeutic target. Pre-clinical studies have shown that adrenoceptor blockade can improve cardiac function but the mechanisms of action within right ventricular (RV) myocytes are unknown. We tested whether the β_1_–adrenoceptor blocker metoprolol could improve RV myocyte function in an animal model of PAH, by attenuating adverse excitation-contraction coupling remodeling. PAH with RV failure was induced in rats by monocrotaline injection. When PAH was established, animals were given 10 mg/kg/day metoprolol (MCT + BB) or vehicle (MCT). The median time to the onset of heart failure signs was delayed from 23 days (MCT), to 31 days (MCT + BB). At 23 ± 1 days post-injection, MCT + BB showed improved *in vivo* cardiac function, measured by echocardiography. RV hypertrophy was reduced despite persistent elevated afterload. RV myocyte contractility during field stimulation was improved at higher pacing frequencies in MCT + BB. Preserved t-tubule structure, more uniform evoked Ca^2+^ release, increased SERCA2a expression and faster ventricular repolarization (measured *in vivo* by telemetry) may account for the improved contractile function. Sarcoplasmic reticulum Ca^2+^ overload was prevented in MCT + BB myocytes resulting in fewer spontaneous Ca^2+^ waves, with a lower pro-arrhythmic potential. Our novel finding of attenuation of defects in excitation contraction coupling by β_1_–adrenoceptor blockade with delays in the onset of HF, identifies the RV as a promising therapeutic target in PAH. Moreover, our data suggest existing therapies for left ventricular failure may also be beneficial in PAH induced RV failure.

## Highlights

• β_1_–adrenoceptor blocker metoprolol delayed the onset of right heart failure in PAH rats.
• Metoprolol attenuated repolarisation remodelling, measured in vivo by telemetry.
• Metoprolol attenuated t-tubule remodeling in single right ventricular myocytes.
• Metoprolol attenuating adverse Ca^2+^ handling remodeling in right ventricular myocytes.
• β–adrenoceptor blockade may be a useful additional treatment for PAH.

## 1 Introduction

Pulmonary artery hypertension (PAH) occurs when the resistance of the pulmonary vasculature increases, resulting in chronic elevated afterload and subsequent right ventricle (RV) dysfunction. Available therapies aim to reduce afterload by promoting vasodilation, however there is no cure and novel treatments are needed [7,21]. RV failure is the most common cause of death in PAH patients, but there is no treatment that specifically addresses RV dysfunction [17]. Despite differences in the structure and function of the RV and left ventricle (LV), it has been proposed that established treatments for LV failure may be beneficial to the failing RV in PAH [21].

β-adrenoceptor blockers (BB) were once considered paradoxical but quickly revolutionized the treatment of LV failure by delaying ventricular remodeling and reducing mortality [6,14]. However, concerns exist over the safety or benefit of BB in PAH [31,34,42], and as such they are not recommended [17,32]. However, PAH patients may receive BB for co-morbidities and recent small scale clinical trials have demonstrated BB safety [1,19,39,40,11]. Pre-clinical studies have shown BB are protective in animal models of PAH, by improving cardiac capillary density, reducing inflammation and fibrosis [4,12,33]. However, the effect of BB treatment on the function of RV myocytes from PAH animals has not been studied. The purpose of our study was therefore to investigate the mechanisms by which improvements, in response to BB treatment, may occur in the PAH myocardium.

Because of the myocardial focus of our study, we chose a β_1_-selective blocker (metoprolol) as this was predicted to target the myocardium more selectively than carvedilol, which has mixed α and β adrenoceptor actions [4] or nebivolol, which has intrinsic vasodilatory pulmonary and systemic properties [33]. Metoprolol is a commonly prescribed BB which reduces mortality in LV failure [44].We used the monocrotaline (MCT) rat model of PAH as it is well established for the study of RV failure [4,12,33,3,15,41]. MCT rats were treated daily with metoprolol (MCT + BB) once PAH was established, to mimic the late detection and treatment that usually occurs in human PAH.

Cardiac excitation contraction coupling plays a fundamental role in pump function and defective Ca^2+^ handling and electrical remodeling reduce contractility and increase electrical instability in RV failure [3,15,41]. We tested whether actions of BB on the RV myocardium attenuated Ca^2+^ handling and electrical abnormalities to facilitate enhanced RV function.

Chronic treatment with metoprolol delayed the onset of RV failure signs in MCT rats, improved RV function and attenuated repolarisation remodeling *in vivo*. There was reduced hypertrophy of RV myocytes. Metoprolol improved the Ca^2+^ handling and contractility of isolated RV myocytes from MCT rats, this was associated with preservation of transverse (t)- tubule morphology, more homogenous Ca^2+^ release and decreased susceptibility to (potentially arrhythmogenic) spontaneous diastolic Ca^2+^ release. These data suggest the beneficial effects of BB in PAH include actions on the mechanical and electrical activity of RV myocytes and that RV treatment may be a valid addition to PAH therapy.

## 2 Materials and methods

See online supplementary material for detailed methods. Experiments were conducted with local ethical approval in accordance with UK Home Office, European Parliament Directive 2010/63/EU guidelines on the use of animals in research.

### 2.1 Animal model and β-blocker treatment

**Fig. 1:**
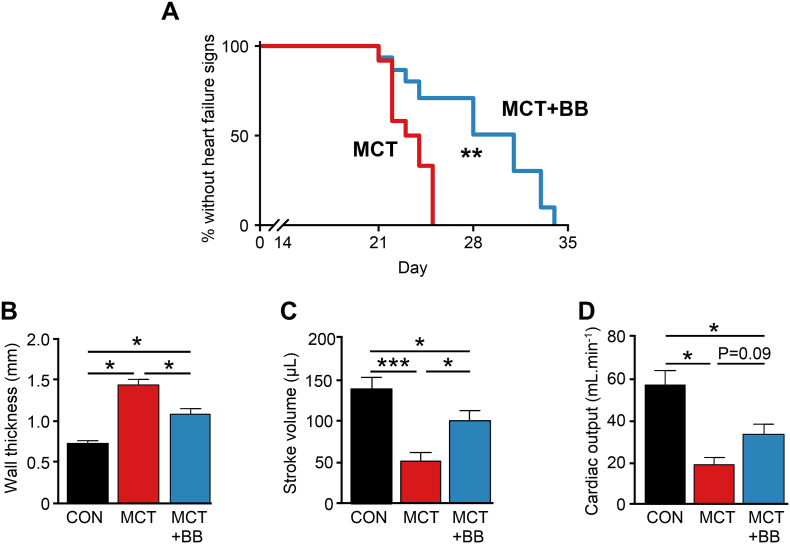
Metoprolol delayed the onset of heart failure in rats with pre-existing PAH. **A** The median day to failure signs was increased from 23 to 31 days post MCT injection (*N* = 12 MCT, 15 MCT + BB rats, ** *P* < .01, Mantel-Cox test). Echocardiography measurements of RV function were performed on the day of heart failure signs (MCT) or the median survival day of MCT rats (CON and MCT + BB). **B** RV wall thickness was reduced in MCT + BB rats compared to MCT, indicating attenuation of hypertrophy. **C** Stroke volume was increased by BB **D** Cardiac output was not significantly increased with BB (*P* = .09 vs MCT). **B-D***N* = 9 CON, 7 MCT, 5 MCT + BB rats. One-way ANOVA. * *P* < .05, *** *P* < .001.

A 3-group experimental design was used, as employed previously by other groups [4,12,33]. Male Wistar rats (200 g) received a single intraperitoneal injection of 60 mg/kg MCT to induce PAH or an equivalent volume of saline. Voluntary oral treatment with a sucrose solution (vehicle) to saline (CON) or MCT-treated (MCT) animals or with 10 mg/kg/day metoprolol dissolved in sucrose solution (MCT + BB) was initiated 15 days post-injection when PAH was established (Table S1). The chosen dose was within the human equivalent dose used clinically in LV failure (see supplementary methods). MCT rats were killed by cervical dislocation, following concussion, upon showing signs of heart failure. The primary indication of heart failure was >10 g weight loss compared to the previous day or weight loss on consecutive days, accompanied by other indicative signs including dyspnoea, piloerection, lethargy and cold extremities [18,27,30,37]. CON rats were killed on the median (±1) survival day of MCT rats. MCT + BB rats used for the survival study (see Fig. 1A) were killed when heart failure signs developed. In other experiments MCT + BB rats were killed on the median (±1) survival day of MCT rats unless heart failure signs occurred earlier.

### 2.2 In vivo monitoring

Transthoracic RV echocardiography was performed under 1.5% isoflurane anesthesia prior to the first dose of BB or sucrose only treatment on day 15, and again on the terminal day. To monitor ECGs and activity in conscious, unrestrained animals, rats were implanted with telemetric probes under anesthesia (2–3% isoflurane) using aseptic techniques. Postoperative analgesia was given by subcutaneous injection of Buprenorphine (Vetergesic 0.1 mg/kg) with the antibiotic Baytril (20 mg/kg).

### 2.3 Histology

10 μm thick transverse cryosections of the RV free wall were stained with fluorescein-conjugated lectin. Myocytes were manually identified in lectin-stained sections by their characteristic oval-shape when cut in cross section. The interior cell boundary of cells was manually identified and traced by an experimenter blinded to the group condition.

### 2.4 Single myocyte studies

Single ventricular myocytes were isolated by enzymatic digestion according to [28] and modified to allow isolation of separate RV and LV myocyte populations. Intracellular Ca^2+^ was measured in myocytes either loaded with 2 μmol/L Fura-4-AM for whole cell epi-fluorescence at 37 ± 1 °C or 6 μmol/L Fluo-4-AM for confocal experiments at 22 ± 1 °C. Sarcomeric shortening was measured using IonWizard software (Ionoptix, Milton USA) during external field stimulation. Spontaneous Ca^2+^ sparks were recorded in Fluo-4 loaded cells using confocal microscopy in linescan mode (188 lines/s, pixel size 0.2 μm). The sarcolemma and t-tubule system of isolated myocytes was stained using di-8-ANEPPS (5 μmol/L) and visualized using confocal microscopy. Sodium-calcium exchange (NCX) current was recorded in discontinuous voltage clamp as the tail current at −80 mV elicited by a 20 ms depolarizing step from −80 mV to +10 mV, following 300 ms priming pulses.

### 2.5 Measurement of mRNA by RT-PCR and protein by Western blotting

Total RNA extraction from RV homogenates was performed using a Qiagen mini-kit. Transcript expression was normalized to the housekeeping genes 18S and GAPDH in the test samples and then made relative to the normalized signal level in the corresponding calibrator sample.

Myocardial tissue was prepared for Western blotting as described previously [15] and probed for amphiphysin II (BIN-1), junctophilin 2 (JP2), and Ca^2+^-handling proteins phospholamban (PLN) and sarco(endo)plasmic reticulum Ca^2+^ ATPase 2A (SERCA) and normalized to GAPDH.

### 2.6 Statistics

Data are presented as mean ± s.e.m. Details of all statistical tests are given in the supplementary methods. *P* < .05 was considered statistically significant. The term, intermediate, is used when comparison of CON vs MCT yields a statistical difference and data mean for MCT + BB has moved towards that for CON such that MCT + BB is statistically different to neither CON nor MCT.

## 3 Results

### 3.1 BB delayed the onset of heart failure

The development of heart failure signs was delayed in MCT + BB rats compared with MCT rats (Fig. 1A). Signs presented with a median of 23 days in MCT rats, whereas using the same criteria, signs presented with a median of 31 days in BB treated rats (*P* < .01 vs MCT). Although BB delayed the onset of heart failure, once developed, the phenotype of failing MCT and failing MCT + BB were not different.

### 3.2 Effect of BB treatment on RV function and electrical remodeling in vivo

The presence of PAH and RV hypertrophy and dilation in MCT treated animals, prior to BB treatment, was confirmed by *in vivo* echocardiography (Table S1). Effective sympathetic blockade by BB treatment was demonstrated by a lack of physical activity-related increase in heart rate, measured by telemetry in freely moving rats (Fig. S1). To assess whether BB improved RV function, CON and MCT + BB animals were studied 23 ± 1 days after a single injection of saline or MCT and compared to vehicle treated MCT rats on the day heart failure signs developed.

MCT animals displayed characteristic increases in lung:body weight RV:body weight and RV:LV + septum weights compared to CON animals (Table S2). Echocardiography (Fig. S2) revealed increased pulmonary artery pressure, RV free wall thickness and increased RV dilatation in MCT rats compared to CON (Fig. 1B, Table S3). MCT rats had decreased RV stroke volume (Fig. 1C, Table S3) and depressed cardiac output (Fig. 1D, Table S3). BB treatment reduced RV wall thickness and increased SV (*P* < .05) compared with MCT, although cardiac output was not significantly increased (*P* = .09, Fig. 1B–D). A 10–15% reduction in mean RV:LV + septum ratio, pulmonary artery pressure and RV internal diameter compared to MCT animals, was not statistically significant (Table S2, Table S3). Lung weights were not changed by BB treatment (Table S2).

**Fig. 2:**
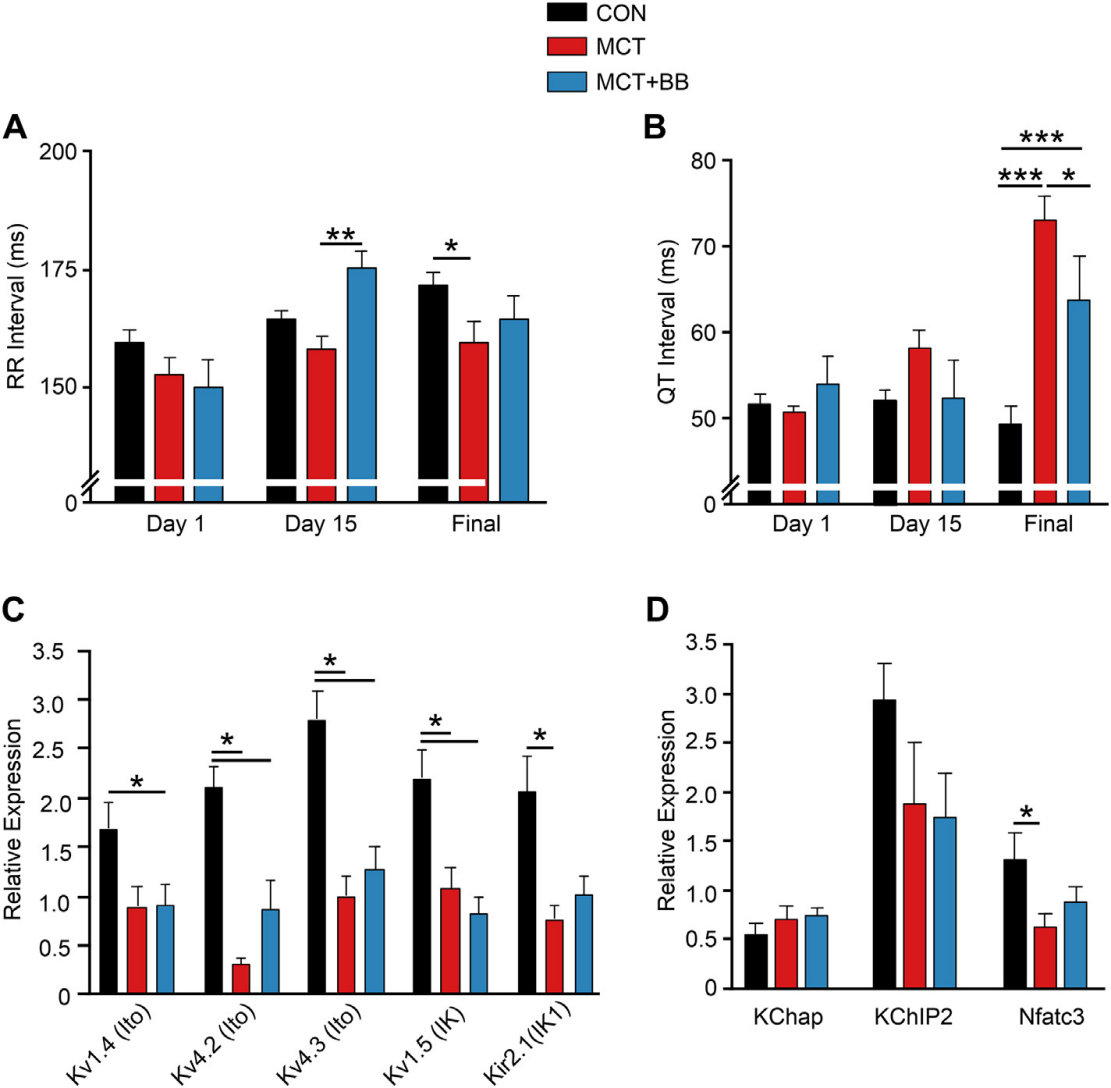
BB attenuated electrical remodeling. Telemetric recording of ECGs during the light (inactive) period on day 1, day 15 (prior to vehicle or BB treatment) and final experimental day. **A** On the final day there was a decrease in RR interval in MCT animals compared to CON, this effect was attenuated by BB treatment. **B** BB treatment significantly reduced the QT prolongation seen in MCT. **C** Final day mRNA levels for channels carrying repolarising K^+^ currents were significantly reduced in MCT compared with CON. For the main channel carrying I_K1_ there was intermediate expression in MCT + BB animals for Kir2.1. **D** Final day mRNA levels for K^+^ channel subunits KChap and KChIP2 did not vary between groups but the expression of the Kv regulator Nfatc3 was reduced in MCT compared with CON, while MCT + BB hearts showed intermediate levels. **A, B***N* = 9–12 animal in each group, two-way ANOVA. **C, D***N* = 10 animals in each group, one-way ANOVA. *P < .05, **P < .01, ***P < .001.

Telemetric measurement of ECGs in conscious unrestrained animals during their inactive period on the final experimental day showed a decrease in RR interval (*P* < .05) and increase in QT interval (*P* < .001) in MCT compared with CON, both these effects were attenuated by BB treatment (Fig. 2A, B).

Prolongation of QT interval represents a longer repolarization and consistent with this, we observed decreased mRNA expression of genes encoding for repolarizing K^+^ channels (I_to_, I_K_ and I_K1_) in MCT compared to CON (Fig. 2C). Expression of the Kir2.1 gene for IK_1_ was decreased in MCT (*P* < .05) but not (*P* > .05) in MCT + BB. Mean mRNA expression for both Kv4.2 and Kv4.3 (responsible for I_to_) were decreased in MCT and MCT + BB. Expression of mRNA for K^+^ channel sub-units/chaperones KChap and KChip2 was not changed but the expression of I_to_ regulator Nfat3 was decreased in MCT (*P* < .05) but not (P > .05) in MCT + BB (Fig. 2D).

### 3.3 BB decreased RV myocyte hypertrophy

**Fig. 3:**
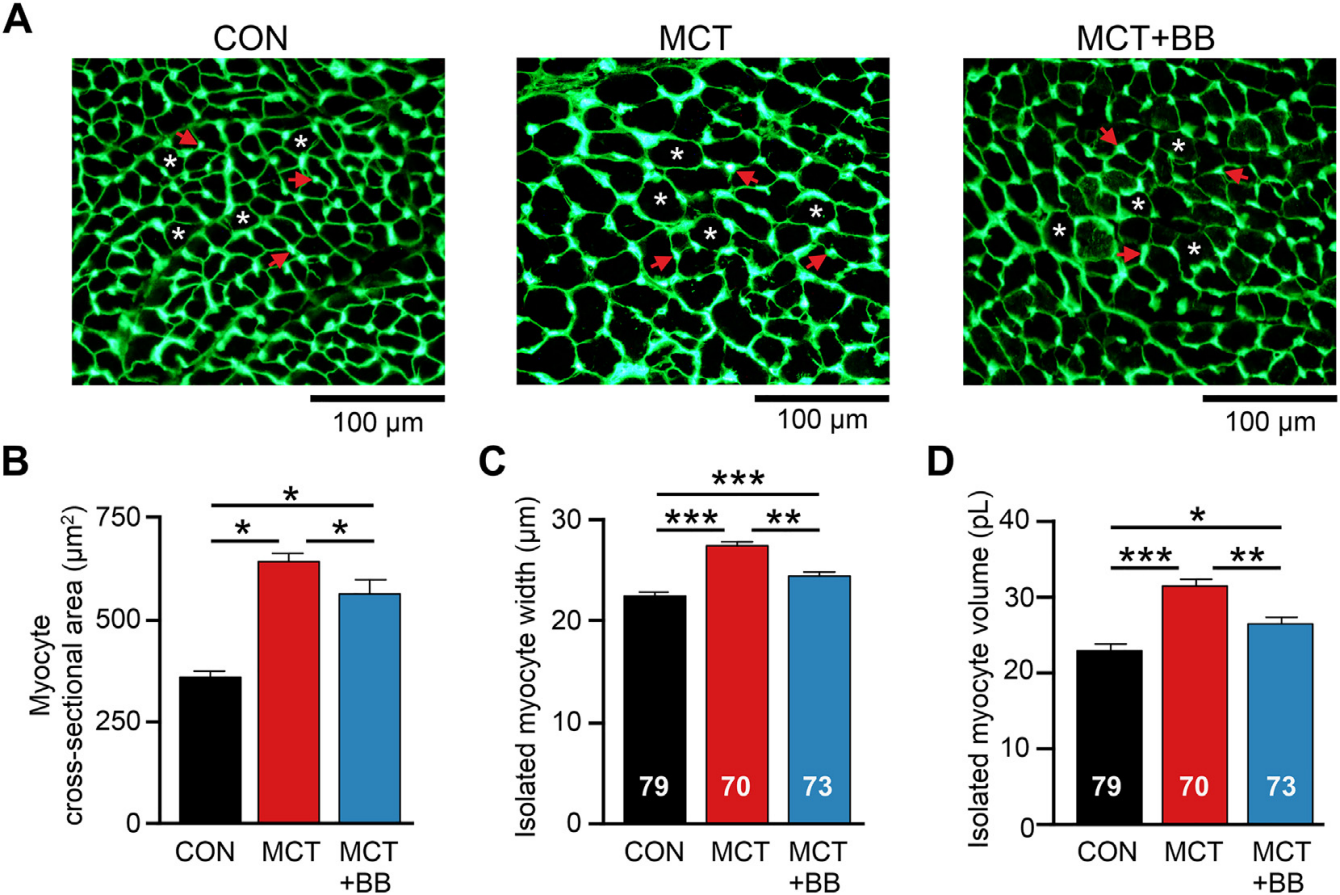
BB attenuated RV myocyte hypertrophy. **A** Exemplar images of RV cryosections stained with lectin to fluorescently label the membrane of myocytes. Capillaries are also stained by lectin and appear as dense spots of labelling. A selection of myocytes (asterisks) and capillaries (arrows) are indicated. **B** Mean myocyte cross sectional area was greater in MCT compared to CON and MCT + BB. The mean value of 2–5 RV sections from each heart were used for statistical analysis. Data represents means from *N* = 6 rats per group. **C** The cell width and **D** cell volume of isolated myocytes was greater in MCT than CON and MCT + BB. **B** N = 6 rats per group, **C,D** N = 6–9 rats per group, myocyte numbers given in the figure. One-way ANOVA. *P < .05, **P < .01, ***P < .001.

Fig. 3A shows exemplar sections of RV showing myocyte boundaries demarcated by fluorescent lectin boundaries. Consistent with measures of RV wall thickness (Fig. 1B), RV myocyte cross sectional area was greater in MCT than CON and MCT + BB (reduced with BB treatment by 12%, P < .05 vs MCT, Fig. 3B). To support this observation, we also measured the geometry of single isolated RV myocytes. RV myocyte width and volume were greater in MCT cells, in comparison dimensions in MCT + BB cells were reduced by 11% and 16%, respectively (Fig. 3C&D). The width and volume of LV myocytes were not different between groups (Table S4).

### 3.4 BB improve RV myocyte contractile response to increased demand

**Fig. 4:**
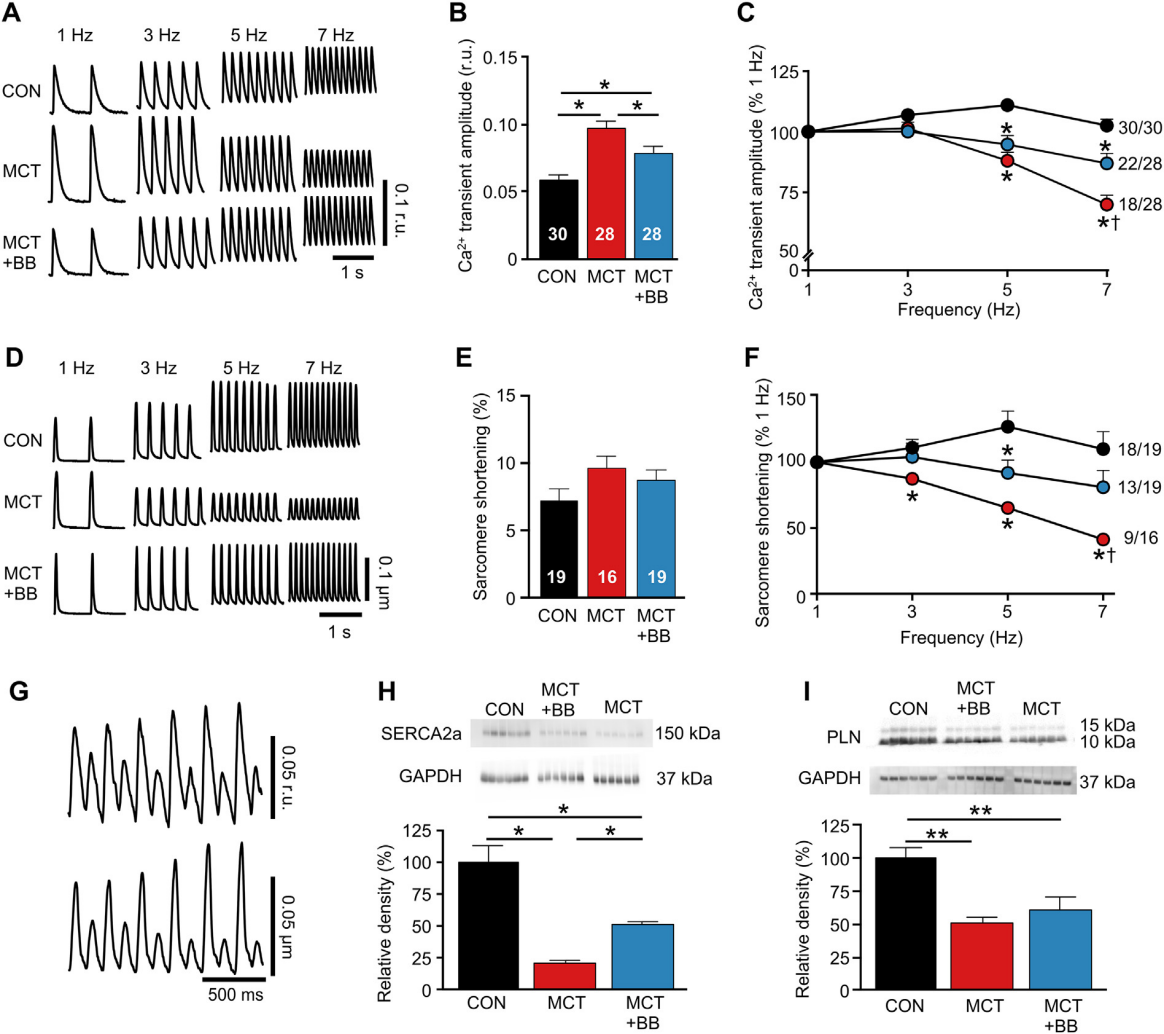
BB improved RV myocyte contraction-frequency relationship. **A** Representative traces of Ca^2+^ transients at different stimulation frequencies. **B** Ca^2+^ transient amplitude of RV myocytes field stimulated at 1 Hz was greater in MCT than CON and MCT + BB. **C** The Ca^2+^ transient amplitude remained stable in CON cells as pacing frequency was increased, whereas it progressively declined in MCT. Ca^2+^ transient amplitude was greater in MCT + BB than MCT at 7 Hz. The number of cells that could be paced at 7 Hz is shown. **D** Representative traces of unloaded sarcomere shortening in a separate group of cells without Fura-4 at different stimulation frequencies. **E** Shortening at 1 Hz was not different between groups. **F** Changes in sarcomere shortening with increased stimulation frequency reflected the changes in Ca^2+^ transient amplitude. Shortening in MCT + BB cells was greater than MCT at 7 Hz. **G** Example recording of a MCT cell unable to regularly pace at 7 Hz due to the emergence of Ca^2+^ release (upper panel) and sarcomere shortening (lower panel) alternans. **H,I** Representative Western blots of SERCA2a, PLN and GAPDH. Note difference in group orders between blots (upper panels) and data (lower panels). **H** There was a reduction in protein expression of SERCA2a in MCT RV that was partially restored by BB**. I** There was a reduction in protein expression of PLN in the RV of MCT and MCT + BB hearts. *N* = 6 rats per group. **B,E,H,I** one-way ANOVA, **C,F** two-way ANOVA. *P < .05, **P < .01 vs CON, †P < .05 vs MCT + BB.

To investigate whether RV myocyte contractility was improved by BB, Ca^2+^ transients and sarcomere shortening were recorded in RV myocytes paced between 1 and 7 Hz (Fig. 4A,D). At 1 Hz, Ca^2+^ transient amplitude was greater (*P* > .05) in MCT than CON and MCT + BB (Fig. 4B). Ca^2+^ transient amplitude fell steeply at higher pacing frequencies in MCT cells, in contrast to a stable Ca^2+^ transient in CON myocytes. The Ca^2+^ transient of MCT + BB myocytes was significantly greater than MCT at 7 Hz (Fig. 4C). Sarcomere shortening reflected the changes in Ca^2+^ transient amplitude at each pacing frequency (Fig. 4E&F). Some myocytes could not be paced at 7 Hz resulting in the formation of Ca^2+^ transient and mechanical alternans (Fig. 4G): 98% (48/49) of CON cells contracted regularly at 7 Hz, whereas 74% (35/47) of MCT + BB myocytes and 61% (27/44) of MCT myocytes could be paced at 7 Hz (*P* < .001, Chi^2^ test). The contraction-frequency relationship of LV cells was similar between groups (Fig. S3). Compromised Ca^2+^ uptake into the sarcoplasmic reticulum (SR) could impair contraction at higher frequencies and we observed decreased protein expression of the SR Ca^2+^ uptake pump, SERCA in MCT rats. This was partially restored by BB treatment (*P* < .05 MCT vs MCT + BB, Fig. 4H). Expression of the SERCA inhibitory protein PLN was decreased in both MCT and MCT + BB rats compared with CON (Fig. 4I).

### 3.5 BB prevented t-tubule remodeling and enhanced Ca^2+^ release in single RV myocytes

**Fig. 5:**
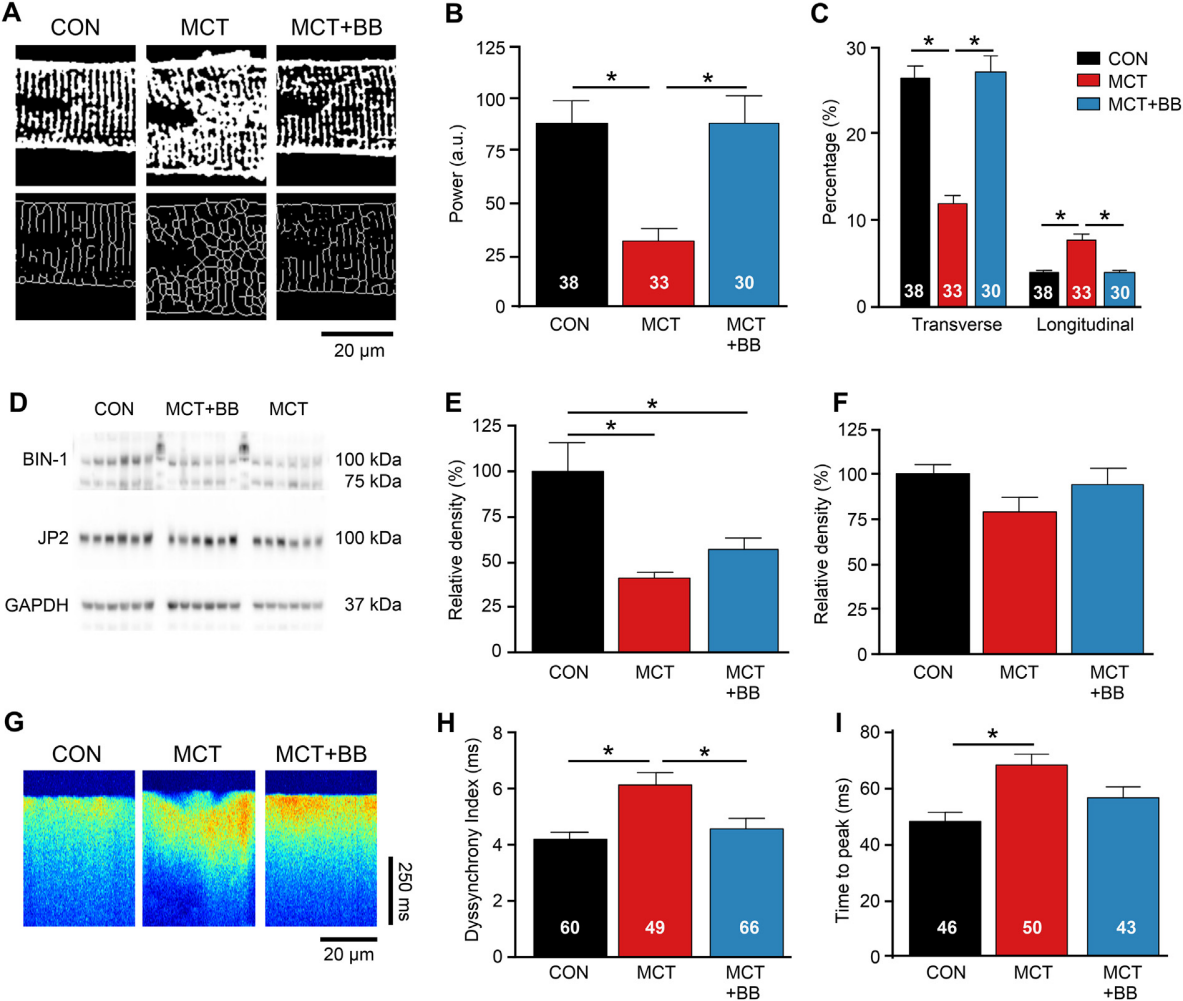
T-tubular structure was preserved and SR Ca^2+^ released synchronised by BB. **A** Binary images of t-tubule morphology (top) and following skeletonization (bottom). **B** The regularity of t-tubules (fast Fourier transform power) was reduced in MCT compared to CON but preserved by BB treatment. Myocyte number given in the fig. **C** Tubule orientation was mainly transverse in CON and MCT + BB myocytes but more longitudinal in MCT myocytes. **D** Representative Western blots of BIN-1, JP2 and GAPDH. Note difference in group orders between blots and data, protein ladders separate group samples for BIN-1. **E** Protein levels of BIN-1 were reduced in MCT and MCT + BB. **F** JP2 was not different between groups. **G** Confocal linescan recordings show spatial non-uniformities in Ca^2+^ transients from MCT cells. **H** Ca^2+^ release was more heterogeneous in MCT cells. **I** Mean time to peak Ca^2+^ was slower in MCT. **B,C**, *N* = 3–4 rats per group, **E,F** *N* = 6 rats per group, **H,I,***N* = 5–8 rats per group. **B,C,F,H,I** one-way ANOVA, **E** non-parametric ANOVA. *P < .05.

T-tubule structure is important for coordinated SR Ca^2+^ release. Fig. 5A shows exemplar confocal images of the t-tubule system (upper panels), and following skeletonization to visualize the predominant direction of tubules (lower panels). It was apparent that tubules in CON myocytes followed a regular striated pattern in the transverse direction, whereas MCT tubules were disorganized, with more longitudinal elements. BB treatment preserved t-tubule regularity, as indicated by greater fast Fourier transform power (Fig. 5B) and predominant transverse orientation (Fig. 5C). T-tubule formation and maintenance is associated with BIN-1 and junctophillin-2 (JP-2) proteins. Fig. 5D shows representative Western blots of BIN-1 and JP-2. BIN-1 expression was reduced in the RV of MCT rats compared to CON (*P* < .05) (Fig. 5E). JP-2 expression was not significantly reduced in MCT compared to CON (Fig. 5F). Fig. 5G shows exemplar Ca^2+^ transients recorded using confocal line scanning, contrasting the uniform Ca^2+^ release in the CON and MCT + BB cells with the scalloped appearance of the MCT Ca^2+^ transient. Dyssynchrony of Ca^2+^ release in MCT cells was reduced by BB treatment (P < .05 vs MCT) (Fig. 5H). The time to peak Ca^2+^ was longer in MCT compared to CON (*P* < .05), but was intermediate in MCT + BB cells (i.e. not significantly different to CON or MCT) (Fig. 5I). Improved t-tubule regularity and more coordinated SR Ca^2+^ release could contribute to the improved contractility of MCT + BB cells.

### 3.6 Attenuation of spontaneous diastolic Ca^2+^ release by BB

**Fig. 6:**
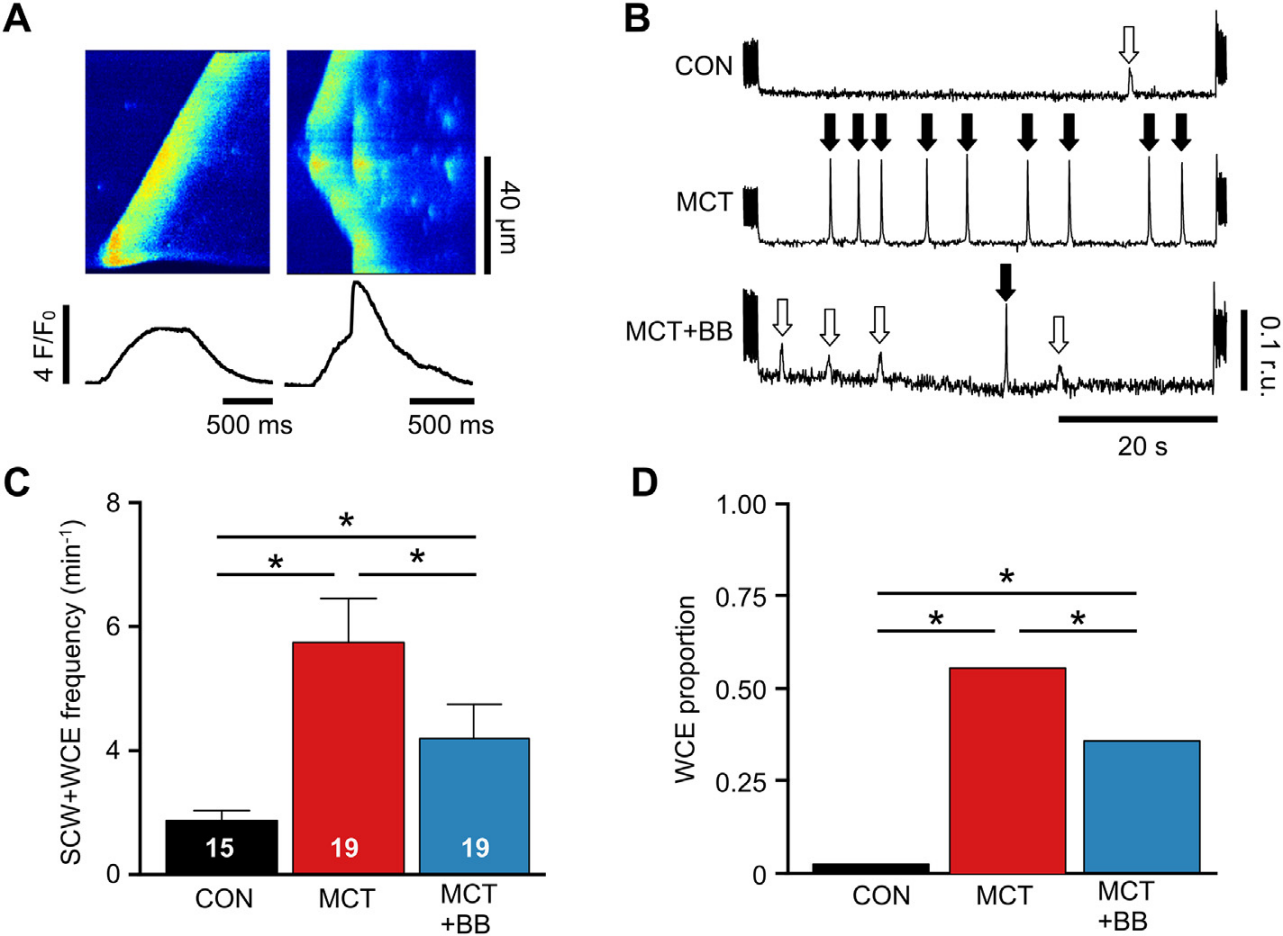
Frequency of spontaneous Ca^2+^ release was decreased by BB. **A** Contrasting morphologies of spontaneous Ca^2+^ waves (SCWs) and whole cell events (WCEs) in linescan (upper traces) and spatially averaged traces (lower traces) from unstimulated MCT RV myocytes. **B** Ca^2+^ release events during a 1 min quiescent period showing SCWs (open arrows) and WCEs (filled arrows). **C**. BB attenuated the total (SCW + WCE) number of Ca^2+^ releases, myocyte numbers given in the fig. **D** BB attenuated the proportion of WCEs. *N* = 5–6 rats per group. **C** one-way ANOVA, **D** Fisher's Exact test. *P < .05.

Spontaneous Ca^2+^ release can provoke arrhythmias. Fig. 6A (left panel) shows a spontaneous Ca^2+^ wave (SCW) propagating across a cell, the average fluorescence in the trace beneath is rounded with a slow upstroke. Fig. 6A (right panel) shows a SCW which triggers a synchronous Ca^2+^ release (termed whole cell event, WCE) throughout the cell. The corresponding line plot is distinguished by a rapid component to the upstroke and larger amplitude. Comparable events were measured with epi-fluorescence in Fura-4 loaded myocytes. The occurrence of SCWs and WCEs was measured in unstimulated myocytes during 60 s rest following 5 Hz stimulation (Fig. 6B). The combined frequency of SCWs +WCEs (Fig. 6C) and the proportion that were WCEs (Fig. 6D) was decreased in MCT + BB myocytes compared with MCT myocytes (*P* < .05).

**Fig. 7:**
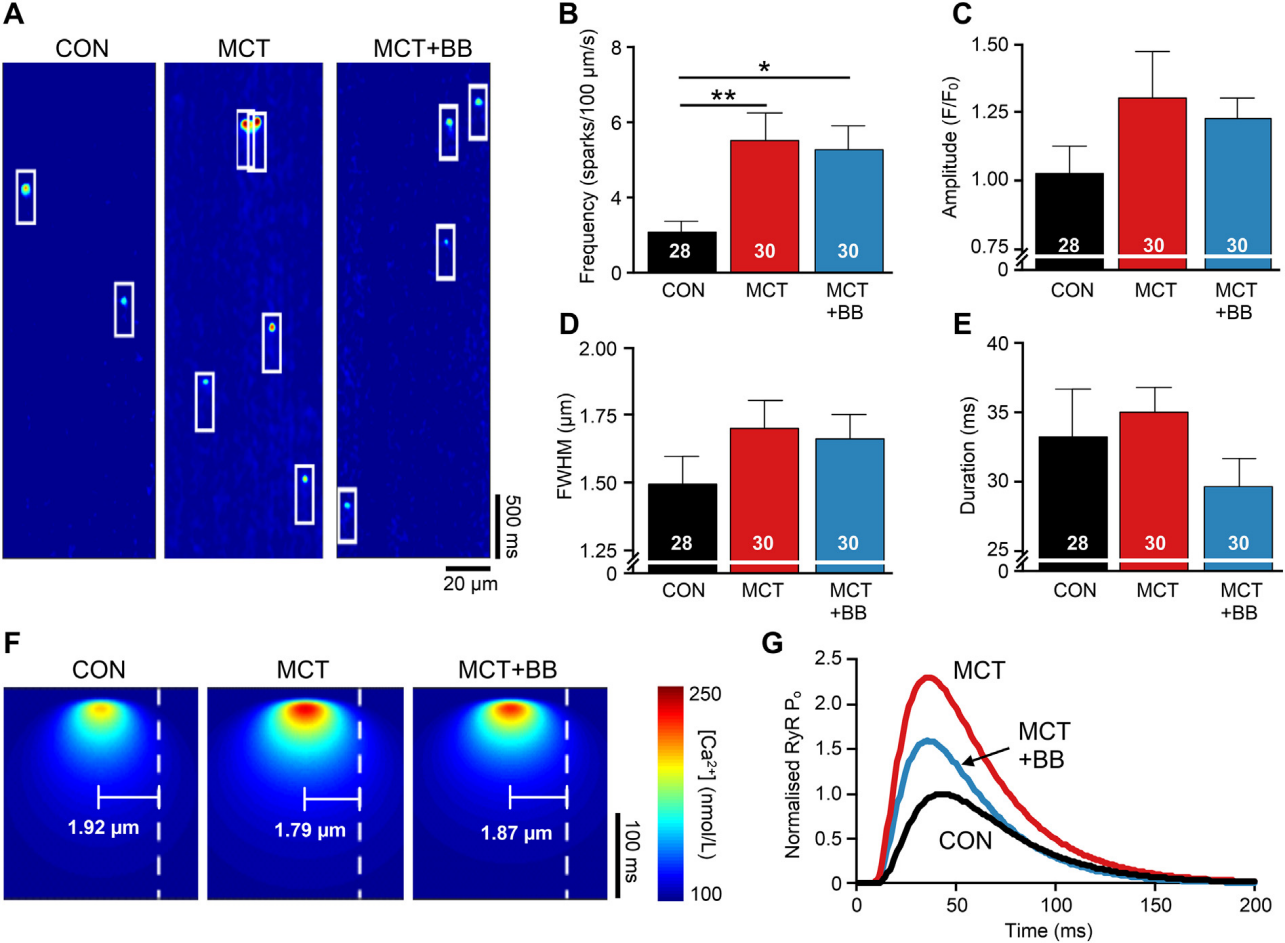
Increased frequency of Ca^2+^ sparks in PAH. **A** Exemplar confocal linescan recordings showing Ca^2+^ sparks (outlined by boxes). **B** Mean Ca^2+^ spark frequency was significantly increased in MCT cells and was not affected by BB treatment. Myocyte numbers given in the figure. There were no statistically significant difference between the mean **C** Ca^2+^ spark amplitude, **D** full width at half-maximum (FWHM) and **E** duration of the 3 groups. **F** To estimate the likelihood of a Ca^2+^ spark activating RyR clusters at a neighbouring junction (i.e. spontaneous Ca^2+^ wave initiation), model Ca^2+^ sparks were created using the mean properties of CON, MCT and MCT + BB Ca^2+^ sparks in (C-E). The cytosolic [Ca^2+^] at distances corresponding to the mean resting sarcomere length of each group (dashed line) was calculated. **G** The peak time-varying RyR open probability (RyR P_o_) in the neighbouring junction was greatest in MCT, but was reduced by BB treatment. Data in (G) is normalised to CON. N = 5–8 rats per group, one way ANOVA. *P < .05, **P < .01.

The precursor to Ca^2+^ waves are Ca^2+^ sparks. Fig. 7A shows exemplar line scan recordings of Ca^2+^ sparks at rest (outlined in white). The mean Ca^2+^ spark frequency was greater in both MCT and MCT + BB RV cells than CON (P < .05, Fig. 7B) without statistically significant changes in other Ca^2+^ spark properties (Fig. 7 C-E). We used a modeling approach to investigate the reasons for the differences in SCW + WCE frequency between MCT and MCT + BB myocytes. A first approximation of the likelihood of Ca^2+^ wave initiation was derived according to the reported sensitivity of rat RyR open probability (Po) to cytosolic [Ca^2+^] [25]. Fig. 7F shows simulated Ca^2+^ sparks constructed from the mean values in Fig. 7C-E. The time-varying RyR P_o_ was calculated at a distance equal to the mean resting sarcomere length of each group from the model spark centroid (white dashed line) (Fig. 7F and Table S4). Fig. 7G shows that modest changes in Ca^2+^ spark properties and a shorter resting sarcomere length increased peak RyR P_o_ in MCT cells by 130% compared to CON. In contrast, the slightly decreased spark size and increased sarcomere length in MCT + BB cells increased RyR P_o_ by only 60% compared to CON. Therefore, despite no change in Ca^2+^ spark frequency, compared with MCT, this simple model predicts that the likelihood of SCW + WCE occurring should be decreased in MCT + BB cells.

**Fig. 8:**
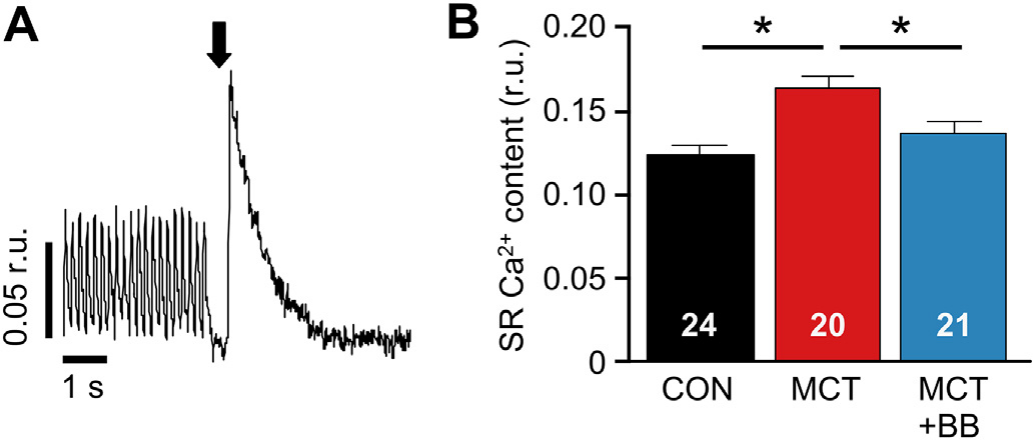
Increased SR load in MCT cells is reversed by BB. **A** SR Ca^2+^ content was estimated during steady state pacing at 5 Hz by rapid application of caffeine (indicated by arrow) to deplete SR stores. **B** SR load was increased in MCT compared to CON, and returned to normal levels by BB. Myocyte numbers given in fig. N = 5–6 rats per group, one way ANOVA. *P < .05.

A greater proportion of Ca^2+^ releases were WCEs in MCT myocytes and this was decreased by BB treatment (Fig. 6D). This effect may be related to a greater SR Ca^2+^ load in MCT myocytes and a normalization of SR Ca^2+^ load by BB treatment, as revealed by rapid exposure to caffeine (Fig. 8). When subjected to the same SR loading protocol MCT myocytes generated greater (P < .05) inward NCX current than CON myocytes (Fig. S4). An increase in SR Ca^2+^ release will generate increased inward NCX current, which may provoke Ca^2+^-induced arrhythmias (see Section 4.4).

## 4 Discussion

Here we show for the first time that β_1_-adrenoceptor blockade attenuated hypertrophy, prevented t-tubule remodeling and improved Ca^2+^ handling in RV myocytes of PAH animals. These effects resulted in an improved contractile response to increased stimulation frequency and less susceptibility to potentially arrhythmic, spontaneous diastolic Ca^2+^ release. We also report the novel finding that β_1_-adrenoceptor blockade attenuated repolarization abnormalities caused by PAH.

Our study was focussed upon the myocardium and for this reason we chose to use a β_1_-adrenoceptor blocker, the major adrenoceptor type in the myocardium. Other BBs have additional actions, e.g. carvedilol, has mixed α and β actions [4] and nebivolol has intrinsic vasodilatory pulmonary and systemic effects [33]. It is possible that these effects may be additionally beneficial in PAH, as vasodilation of the pulmonary vasculature is a central strategy in current PAH therapy.

### 4.1 In vivo cardiac function and electrical remodeling

The improvement in stroke volume and trend for increased cardiac output (*P* = .09) in MCT + BB rats occurred in the absence of significant RV afterload reduction, similar to reports using other BB [4,12,33]. This suggests the protective effects of metoprolol are mediated through direct actions on the myocardium, rather than secondary to pulmonary responses, and are attributable to its β_1_-selectivity. In conscious animals, metoprolol treatment did not reduce resting heart rate, but did block activity-induced increase in heart rate, this is consistent with the effect of bisoprolol reported by [12]. In conscious MCT rats, heart rate was increased compared to CON (Fig. 2A), conversely, under anesthesia, heart rate was decreased in MCT and MCT + BB rats (Table 3), which is consistent with the observations of [3]. This increased sensitivity to anesthesia should be considered when interpreting cardiac output data.

In human PAH patients QT interval prolongation correlated with mortality [35] and we found BB attenuated QT prolongation. BB treatment did not cause statistically significant increases in mRNA expression of the main channels that carry I_to_ (Kv4.2 and Kv4.3) however there was a consistent graded expression (CON>MCT + BB > MCT) of mean mRNA for Kv4.2, Kv4.3 and intermediate expression of both the I_to_ regulator Nfatc3 [36] and the Kir2.1 channel (I_K1_). QT prolongation in MCT animals is linked to decreased expression of Kv channel mRNA, therefore our observations indicate a mechanism whereby QT prolongation may be attenuated by BB via changes in expression of several Kv channels.

### 4.2 Decreased RV hypertrophy with BB

Measurement of RV myocyte size and RV wall thickness by echocardiography indicated a reduction in RV hypertrophy with BB. Although the decrease in RV:body weight with BB treatment was not statistically significant the mean change (11%) was similar to the 15% decrease in myocyte size. By necessity, RV free walls were weighed following exposure to collagenase, prior to dispersion into single myocytes. Differences in the response of tissue to digestion may obscure smaller differences in RV weight between MCT and MCT + BB.

The hypertrophic response to pressure overload is considered a compensatory mechanism to normalize elevated wall stress [20]. However, blocking the hypertrophic response does not necessarily worsen outcomes in LV failure [10,38]. Furthermore, myocardial hypertrophy is associated with increased incidence of death [26], possibly by reducing coronary perfusion, increasing oxygen diffusion distances, or increasing energy expenditure.

### 4.3 BB improved the inotropic response of RV myocytes to increased demand

A definition of heart failure is the inability to cope with increased demand (e.g. the NYHA classification). In the MCT model this presents as a steeply negative contraction-frequency relationship linked to a fall in the amplitude of the Ca^2+^ transient [3,15]. Disruption of the t-system uncouples LTCC and RyR resulting in inhomogeneities in Ca^2+^ release which slow the rate of force development [13]. Loss of t-tubules in human LV failure is associated with reduced contractility of the ventricle [9]. We observed a loss of regularly spaced t-tubules in MCT. T-tubule formation and maintenance is linked to BIN-1 and JP2 [8] and in MCT myocytes we observed a reduction in BIN-1 protein, as reported in LV failure [8], with a trend (mean CON > MCT + BB > MCT) for both BIN-1 and JP2. The downregulation of SERCA in MCT is likely to limit the ability to cycle Ca^2+^ normally and this too was improved by BB. In addition we have previously shown that the contraction-frequency relationship in MCT myocytes is influenced by BB via expression of creatine kinase [15,16]. Repolarization profile can modulate Ca^2+^ handling [5] and we have shown that in MCT myocytes the stimulation frequency dependent fall in contraction is, at least in part, caused by the stimulation frequency dependent fall in action potential duration (APD) [3,22]. Therefore, the prolonged APD and QT interval occurring in the MCT model may facilitate greater SR load through enhanced Ca^2+^ entry, while steeper APD restitution contributes to the steeper stimulation frequency dependent fall in Ca^2+^ transient amplitude [3,22]. Mechanical alternans are a consequence of Ca^2+^ transient alternans (Fig. 4G) which are themselves provoked either by dysfunction of Ca^2+^ uptake/release and/or by APD alternans, see [3]. These responses may be improved by the attenuation of QT prolongation by BB. Thus, cumulative factors influencing myocyte structure, Ca^2+^ handling protein expression, and metabolic and electrical activity are likely to have improved myocyte contractile function.

### 4.4 Improved diastolic Ca^2+^ handling following BB

MCT myocytes produced more total spontaneous Ca^2+^ releases (SCWs+WCEs), with a greater proportion of WCEs; both types of Ca^2+^ release were reduced by BB. RyR are clustered at the sarcomeric Z-line [23] and a shorter SL will bring Ca^2+^ release units of adjacent Z-lines closer together and increase the probability of wave initiation and propagation [24]. For Ca^2+^ sparks to initiate SCW, they must raise cytosolic Ca^2+^ sufficiently to activate RyR clusters ~1.9 μm from the original spark site, since RyR P_o_ is thought to depend on cytosolic [Ca^2+^]^~2.8^ [25]. Assuming isotropic Ca^2+^ diffusion in the cytosol [2], our modelling predicts that the modest changes in Ca^2+^ spark characteristics we observed, together with increased RyR spacing in response to BB are sufficient to decrease RyR open probability and thus the chances of Ca^2+^ wave propagation.

WCEs are thought to be linked to delayed after depolarization (DAD) arrhythmias. Ca^2+^ induced DADs are driven by inward NCX current and the greater the SR load, the greater the potential for Ca^2+^ release and subsequent depolarizing NCX current (Fig. S4). The resultant magnitude of depolarization for a given NCX current density is dependent upon resting membrane stability, which is determined largely by I_K1_ current carried through Kir2.1 channels [29]. We have shown that in MCT myocytes there is an increase in SR load (Fig. 8) and NCX current (Fig. S4) and have recently reported that membrane stability is decreased in these cells [22]. Thus, the decrease in WCEs in response to BB can be explained by the decrease in SR Ca^2+^ load and intermediate level of Kir2.1 expression in MCT + BB myocytes.

### 4.5 Limitations

The MCT model is used extensively to study the effects of PAH on the RV due to the reliable induction of heart failure, although its exact mode of action is not fully understood and vascular remodeling does not lead to plexiform lesions as seen in human patients of PAH, for recent reviews of the model see [18,27,30,37]. We cannot discount systemic effects of BB, however the LV, which was exposed to the same circulating levels of metoprolol as the RV, was unaffected by BB treatment (Table S4 & Fig. S3). Our Ca^2+^ spark model was intended to test the role of Ca^2+^ spark properties and cell geometry on SCW initiation; changes in RyR sensitivity or cytosolic [Ca^2+^] may further influence wave initiation.

### 4.6 Conclusions and Implications

The need to study the specific nature of RV pathophysiology has been highlighted [43]. Notwithstanding this important point, our novel observations show beneficial effects of BB on RV structural, mechanical and electrical remodelling and on intracellular Ca^2+^ handling. Despite the different structure and operating environments of the RV and LV, existing treatments for LV failure may be useful adjuncts to therapies targetting pulmonary vasculature dysfunction in RV failure resulting from PAH.

## Funding

This work was supported by British Heart Foundation grant [PG/13/3/29924].

## Conflict of interest

None.

## Disclosures

Some data from Fig. 1A and Table S4 have been reported in symposium paper format [16].

## Appendix A Supplementary data

Supplementary material Image 1
