# Supplementary material for: Beta1-adrenoceptor antagonist, metoprolol attenuates cardiac myocyte Ca2+ handling dysfunction in rats with pulmonary artery hypertension
Source: J Mol Cell Cardiol. 2018 Jul;120:74–83. doi: 10.1016/j.yjmcc.2018.05.015 (PMC6013283; doi:10.1016/j.yjmcc.2018.05.015)
Supplement: Supplementary file 1 — Supplementary material [file mmc1.pdf]

Supplementary Material

Supplementary Methods

Monocrotaline model of pulmonary artery hypertension

All experiments were conducted in accordance with the Animals (Scientific Procedures) Act (1986), European Parliament Directive 2010/63/EU and local ethical approval. Monocrotaline (MCT; Sigma, UK) was prepared fresh on the day of injection by dissolving 68 mg MCT in a small volume of 1 M HCl with 140 mM NaCl, pH was adjusted to 7.4 with 5 N NaOH, the final concentration of MCT was 20 $mg.mL^{-1}$. Male Wistar rats bred at the University of Leeds weighing 200 ± 20 g were given an intraperitoneal injection of 60 mg/kg MCT to induce PAH, or an equivalent volume of 140 mM saline (CON). Milli-Q grade water was used for all experiments. Rats were housed three per cage at 20-22°C in 50% humidity on a 12 h light/dark cycle with *ad libitum* access to food and drinking water. Rats were weighed and checked for signs of heart failure twice per week from the day of MCT injection (day 0) then daily from day 15 onwards. Animals treated with MCT and vehicle were termed MCT, animals treated with MCT and metoprolol were termed MCT+BB. MCT animals were killed upon showing signs of heart failure (weight loss on consecutive days, dyspnea, piloerection). These signs are standard for this model and been validated against *in vivo* measures confirming RV failure e.g. (Nogueira-Ferreira *et al.*, 2015;Ryan *et al.*, 2013;Maarman *et al.*, 2013;Gomez-Arroyo *et al.*, 2012;Hardziyenka *et al.*, 2006), Table S2. CON and MCT+BB rats were killed on the median (±1) final day of MCT rats with the exception of survival to heart failure signs studies (Fig.1A) when MCT+BB animals were killed upon showing heart failure signs.

Treatment with metoprolol

All (saline and MCT injected) rats voluntarily ingested vehicle solution 20% (v/v) Ribena® and 10% (w/v) sucrose from a 1 mL syringe. Treatment with metoprolol tartrate (10 mg/kg/day; Santa Cruz Biotechnology, TX) was initiated 15 days after MCT injection (MCT+BB), when PAH and hypertrophy are already established (Table S1). Metoprolol was dissolved in vehicle at a concentration of 1.25 mg/mL, which was found to be the highest concentration rats would willingly consume. Voluntary consumption avoided stressful daily gavage or injections. Dosing was administered within 2 h prior to the start of the dark (active) cycle. Daily dose volume was approximately 2.5 mL and adherence was calculated from any remaining unconsumed solution in the syringe. Metoprolol and vehicle solutions were prepared freshly every 2 days and covered in aluminium foil to prevent photo inactivation of metoprolol.

The human equivalent dose (HED) was calculated by multiplying the animal dose by the ratio of animal to human mass per body surface area (K_m), where K_m is the empirically derived mean body weight (kg) divided by body surface area (m^2):

$$HED\ (mg/kg) = Animal\ dose\ (mg/kg) \times \frac{Animal\ K_m}{Human\ K_m}$$

We treated rats with a dose of 10 mg/kg/day metoprolol, the K_m for rats and adult humans is 6 and 37 kg/m^2, respectively (Reagan-Shaw *et al.*, 2008). The human equivalent dose of metoprolol used in our study was 1.62 mg/kg/day, within the range of typical starting and target clinical doses of 0.17 to 2.67 mg/kg/day for a 75kg patient

Echocardiography

Rats were anesthetized in a Perspex chamber ventilated with 5% isoflurane (IsoFlo, Abbott Laboratories, IL) mixed with medical O_2. Anesthesia was confirmed by the lack of pedal-withdrawal reflex in response to a toe-pinch. Rats were transferred to a heated platform and secured in a supine position with pieces of autoclave tape over the paws. Anesthesia was maintained under ~1.5% isoflurane and adjusted according to breathing frequency. The chest was shaved with electric clippers and depilatory cream applied to remove remaining hair. For recovery experiments, rats were allowed to awaken on the heating pad while breathing medical O_2.

Serial, non-invasive transthoracic echocardiography was performed on anesthetized rats using a Vivid7 ultrasound machine (GE Healthcare, WI) with a curvilinear 11.5 MHz 10s probe (GE Healthcare, WI). Ultrasound gel (Cardiacare, Essex, UK) was applied to the shaved chest, taking care to avoid air bubbles. The probe was lightly pressed against the chest and oriented to view the heart in transverse cross-section. Geometry of the RV and LV were measured using brightness (B-) and motion (M-) modes taken from the parasternal short axis (PSAX) view at the level of the LV papillary muscles. Imaging depth was set to 2 cm with data recorded at 100 Hz. Care was taken to avoid reflections and shadows in the viewing window due to the lungs and ribs.

Two-dimensional B-mode and one-dimensional M-mode measurements were taken when chamber area or diameter was greatest in diastole and smallest during systole. RV and LV areas were measured in B-mode by tracing the endocardium of the ventricles. M-mode images were constructed by stacking the pixel values along a line bisecting the heart through the RV, septum and LV as a function of time. Wall thickness, chamber diameter and pulmonary artery diameter (PAD) were measured in M-mode.

Pulmonary artery (PA) blood flow was measured using Doppler ultrasound taken from the PSAX view near the base of the heart at the level of the pulmonary arch. When the angle of incidence between sound waves and direction of blood flow is <20° it has minimal influence on the calculated velocity (Feigenbaum, 2004). Blood flow was measured in a 1 mm sample volume placed 1-2 mm from the PA valve leaflets in a region of the PA with maximal laminar flow, therefore no angle correction was applied. Pulmonary artery acceleration time (PAAT) was measured from the onset to the peak of systolic flow. Systolic RV pressure was predicted from linear regression of PAAT vs invasive RV catheterization (Jones *et al.*, 2002). The resulting equation of the line was RV Pressure (mmHg) = 117.2 – 1.828 x [PAAT] ($P<0.001$, $R^2 = 0.58$). The PA velocity-time integral (VTI) was measured from the start to the end of systolic flow. Cardiac output was calculated by multiplying stroke volume (SV) by heart rate. RV. SV was calculated from the VTI and PAD (Rudski *et al.*, 2010). Assuming a circular PA cross-sectional area:

$$SV\ (\mu L) = VTI\ (cm) \times \left(\frac{PAD\ (cm)}{4}\right)^2 \times 1000$$

Each echocardiography examination was assigned a unique identifier consisting of a computer-generated 5-digit random number, allowing image analysis to be performed blinded to the group and time point.

Telemetric monitoring of ECG and activity in conscious, unrestrained animals

Telemetric devices (TA10ETA-F20, Data Sciences, St. Paul, MN) were surgically implanted under anesthesia (2–3% isoflurane) using aseptic techniques. Postoperative care involved administration of an analgesic by subcutaneous injection of Buprenorphine (Vetergesic 0.1 mg/Kg) and subcutaneous injection of an antibiotic (Baytril 20mg/kg). The chosen devices are designed for mice, making them smaller and lighter than standard rat devices, and were used to minimize recovery time and the effects of implantation. These devices recorded ECG and activity. ECGs were recorded in conscious, unrestrained single-caged rats during light and dark cycles (following a standard 12:12h light-dark regime). The data was acquired using Dataquest A.R.T. 4.1 Gold software (Data Sciences) and the ECG traces were

analyzed using Chart 7 ECG analysis module (AD Instruments) using the pre-set rat ECG configuration which accommodates for the absence of a ST interval. For a given animal the ECG characteristics are the average of approx. 20,000 cycles. In 24h recordings mean hourly heart rate and activity were calculated.

Myocyte labelling

Hearts were perfused retrograde at a constant flow of 7 mL/min/g with Tyrode solution (IS) containing in mmol/L: 130 NaCl, 5.4 KCl, 0.4 NaH_2PO_4, 5 HEPES, 10 glucose, 10 creatine, 20 taurine, 1.4 $MgCl_2$, pH 7.4 with 5N NaOH) containing 100 μmol/L EGTA to arrest the heart in diastole and clear the coronary circulation of blood. A portion of mid-RV free wall was dissected on ice using a safety razor blade. The tissue was covered in a layer of OCT (optimal cutting temperature; VWR, Lutterworth, UK) and the whole sample inverted and frozen in isopentane, precooled by liquid N_2. Samples were tightly wrapped in PVC film and stored in airtight plastic bags at -80 °C.
Samples were warmed to -20°C in a cryostat (CM1900, Leica, Milton Keynes, UK) and 10 μm thick sections were cut orthogonal to the long axis of the tissue using a microtome. Sections were transferred to poly-L-lysine coated microscope slides (Polysine Microscope Adhesion Slides, VWR, Lutterworth, UK) and marked with a 5-digit computer-generated random number unique to each heart, allowing image acquisition and analysis to be performed blinded to the origin of the sample. Sections were fixed in acetone on ice for 5 min then allowed to air dry and stored at -80°C until use. Slides were incubated in the dark for 1 h with fluorescein-conjugated lectin from *Griffonia simplicifolia* (Vector Laboratories, CA), at a final concentration of 10 μg/mL in PBS. Slides were rinsed in 3 washes of PBS then mounted in an anti-fade mounting medium (Vectashield, Vector Laboratories, CA) diluted 1:8 in PBS, sealed with a coverslip and stored in the dark until imaging (within 24 h). Slides were imaged using epifluorescence on a Nikon Eclipse E600 microscope with a Nikon Plan Fluor 20X objective. A 0.15 mm^2 area (~200-400 capillaries per field) was imaged at a resolution of 2560x1920 pixels using a camera (MicroPublisher 5.0, QImaging, Surrey, Canada) attached to the side port of the microscope. An LED lightsource provided excitation light which was filtered through a 487±10 nm bandpass filter and fluorescence collected through a 533±20 nm bandpass filter. Only areas cut in good transverse cross-section were analysed. Lectin binds to the glycocalyx of myocytes allowing the cell membrane to be identified, while leaving the interior of the cell negatively stained. Other cell types such as fibroblasts and capillary endothelial cells may also be stained by lectin, however the interstitial localization of fibroblasts and the small size of capillaries (~2 μm radius) permitted these to be excluded from analysis. 2-5 sections were analysed for each heart and the results averaged to give a representative value for each animal; this representative value was used for subsequent statistical analysis.

Right ventricular myocyte isolation

Isolated hearts were cannulated on a Langendorff perfusion system and perfused retrograde at a constant flow of 7 mL/min with a I.S. supplemented with 750 μmol/L Ca^{2+} until the coronary circulation cleared of blood and spontaneous sinus rhythm was established. Perfusion was switched to I.S. containing 100 μmol/L EGTA for 4 min to arrest contraction. The heart was then perfused with 40 mL I.S. containing 50 μmol/L Ca^{2+}, 1 mg/mL collagenase Type II (activity 270 U/mg, Worthington Biochemical, New Jersey, USA) and 0.1 mg/mL protease (Type XIV, Sigma, UK). Enzyme solution was collected and recirculated for a total digestion time of 7 min.
Following Langendorff perfusion, the atria were removed and the ventricles split into RV free wall and LV portions. These 2 pieces of tissue were then separately, coarsely minced and placed into different 4 mL enzyme solution aliquots, to allow distinct RV and LV myocyte populations to be isolated. The tissue was gently agitated in a water bath at 37 °C for 4 min using a rotary shaker to liberate isolated

cardiomyocytes. The tissue and enzyme solution were strained through nylon mesh (200x200 µm) and the enzyme solution centrifuged at 50 x g for 40 seconds. The supernatant was removed and the cell pellet resuspended in 750 µmol/L Ca^{2+} I.S. Cells were stored for up to 8 h at room temperature.

Recording of sarcomere shortening and cytosolic Ca^{2+} in myocytes

A drop of cells was placed in the perfusion chamber on the stage of an inverted microscope (Eclipse TE300, Nikon, Japan) and viewed under a 40x objective (Nikon, Japan). Local perfusion of a single cell was achieved using an 8-channel pipette (MPRE8, Cell MicroControls, Norfolk, VA). Bulk and local perfusion temperature were controlled independently using a TC^2_{bip} bipolar temperature controller (Cell MicroControls, Norfolk, VA). Experimental Tyrode solution contained, in mmol/L: 137 NaCl, 5.4 KCl, 0.33 NaH_2PO_4, 0.5 $MgCl_2$, 5 HEPES, 5.6 glucose, 1 $CaCl_2$, pH 7.4 with 5N NaOH. Sarcomere shortening was recorded during electrical field stimulation delivered by two platinum electrodes placed at the sides of the bath. Pacing frequency was controlled between 1-7 Hz with a pulse duration of 5 ms using an analogue stimulator (SD9, Grass Instruments, Warwick, RI).

Rod-shaped, quiescent cells with clear striations were used for experiments. A video image of cells was acquired using a video camera (MyoCam-S, IonOptix, Milton, MA) mounted to the side port of the microscope and displayed on a computer monitor. Cell volume was calculated from cell length, width and depth (assuming cell width varied with depth in the ratio of 1.44, as found by (Natali *et al.*, 2002). Volume was calculated assuming an elliptical geometry with 54% of the volume of a parallelepiped (Satoh *et al.*, 1996). Data were recorded at 250 Hz. Cell length and width were measured using a ruler overlaid on the computer monitor and calibrated with a micrometer graticule (Graticules Ltd, Kent, UK). The mean sarcomere length was calculated online using the SarcLen algorithm in IonWizard software (IonOptix, Milton, MA).

Ca^{2+} epi-fluorescence imaging with Fura-4F

Changes in cytosolic Ca^{2+} were monitored using the cell permeant, acetoxymethyl (AM) ester form of the ratiometric Ca^{2+} indicator Fura-4F (Fura-4F-AM, Invitrogen, UK). Cells were incubated with 2 µmol/L Fura-4F-AM from a 1 mmol/L stock dissolved in DMSO for 20 min at room temperature on a rocker and protected from light to prevent photobleaching. Cells were re-suspended in fresh experimental Tyrode and allowed to de-esterify for 30 min before being used for experiments.

A red filter was placed in the path of the microscope light source and experiments were conducted in the dark allowing the cell to be visualised while minimising photobleaching and fluorescence interference. A cell was centred in the field of view and a rectangular light excluding diaphragm adjusted to fit around the cell boundary. A monochromator (Optoscan, Cairn Research, Kent, UK) alternately illuminated cells with 340±10 and 380±10 nm light. The excitation light source was a 150 W Xenon arc lamp (Cairn Research, Kent, UK). Returning light was directed through a dichroic mirror which separated light >600 nm towards the video camera for cell viewing and light <600 nm was passed through a 510 ± 20 nm bandpass filter into a photomultiplier tube and measured by a spectrophotometer integrated into the monochromator unit. The resulting fluorescence collected at 510 nm in response to 340/380 nm excitation light was converted to a digital signal and output to IonWizard to calculate and record the 340/380 ratio at 200 Hz. Unloaded shortening was recorded in a separate group of cells without Fura-4F to avoid Ca^{2+} buffering affecting contraction. A similar contraction-frequency relationship was also found in Fura-4 loaded cells (data not shown).

Sarcoplasmic reticulum Ca^{2+} content estimation

Sarcoplasmic reticulum (SR) Ca^{2+} content can be estimated by the amplitude of the caffeine-evoked Ca^{2+} transient (Varro *et al.*, 1993). Cells loaded with Fura-4F were paced at 5 Hz for 1 min in 1 mmol/L Ca^{2+}

Tyrode to standardise SR loading, then stimulation was stopped and the solution quickly switched to Tyrode containing caffeine (20 mmol/L) using local perfusion.

Confocal line scan Ca^{2+} imaging with Fluo-4

The spatiotemporal characteristics of Ca^{2+} release were measured in cells loaded with the non-ratiometric Ca^{2+} indicator Fluo-4-AM, using line scanning confocal microscopy. A Nikon Eclipse TE2000-U confocal microscope with 40x oil immersion objective was used with excitation light provided by a 488 nm sapphire laser scanning at 188 lines per second. Data acquisition was performed using LaserSharp 2000 software (BioRad, UK). Pixel size in XY was 0.2 µm. Fluorescence was passed through a 515 nm longpass filter and recorded using a photomultiplier tube (PMT). Cells were incubated with 6 µmol/L Fluo-4-AM (Invitrogen, UK) from a 1 mmol/L stock in DMSO for 20 min at 22±1°C. Cells were re-suspended in fresh Tyrode and allowed to de-esterify for 45 min at 4°C before experiments. Cells were superfused with 750 µmol/L Ca^{2+} Tyrode at 22±1°C and electrically stimulated to steady state for 30 s at 0.5 Hz with a 10 ms pulse duration (S48, Grass Instruments, Warwick, RI) using a pair of platinum electrodes. Ca^{2+} sparks were detected and analysed using an algorithm based on (Kong *et al.*, 2008), implemented in Matlab. The uniformity of systolic Ca^{2+} release was quantified using the standard deviation (Dyssynchrony Index) of the time to half maximum Ca^{2+} measured line-by-line across the length of the cell (Caldwell *et al.*, 2014).

Ca^{2+} spark and RYR Po modelling

Synthetic model Ca^{2+} sparks were generated in Matlab according to (Kong *et al.*, 2008), using the mean properties (amplitude, width, duration) of Ca^{2+} sparks measured experimentally in CON, MCT and MCT+BB cells. Model sparks were pseudocalibrated into units of $[Ca^{2+}]$ (nmol/L), according to (Cannell *et al.*, 1994):

$$[Ca^{2+}]_i = \frac{KR}{(K/[Ca^{2+}]_{rest}) - R + 1}$$

Where K is the *in vivo* affinity of Fluo-4 for Ca^{2+} (K_d ~1000 nmol/L), R is the self-ratio fluorescence (F/F_0), and $[Ca^{2+}]_{rest}$ is the resting Ca^{2+} concentration (~100 nmol/L) (Wier *et al.*, 1987).

Spontaneous Ca^{2+} release

Spontaneous Ca^{2+} releases recorded in confocal linescan images of Fluo-4 loaded cells occurred either as propagating waves, travelling through the cell without a global Ca^{2+} transient occurring, (spontaneous calcium wave, SCW) or as releases which developed into a synchronised Ca^{2+} transient (whole cell event, WCE). This was apparent in spatially averaged x-t linescan images as a biphasic slow initial upstroke followed by a secondary rapid upstroke and decay. The SCW+WCE frequency at room temperature was low, therefore we recorded Ca^{2+} release in Fura-4 loaded cells at 37°C following pacing at 5 Hz and found similar Ca^{2+} release profiles. WCEs were considered as such if the upstroke velocity was ≥50% that of a systolic Ca^{2+} transient recorded in the same cell. This internally controlled for variation in the rate of Ca^{2+} release between groups (see Fig. 5 in main text). The fluorescence derivative was filtered using a 10 Hz Zero-Phase Lowpass filter to aid wave identification (IonWizard, IonOptix,

Milton, MA). Only cells exhibiting at least one Ca^{2+} release were included in the analysis; 15/24 CON, 19/21 MCT+BB and 19/21 MCT cells exhibited at least one Ca^{2+} wave.

Tubular network imaging

A 1 mmol/L stock solution of di-8-ANEPPS was prepared in DMSO and stored at -20°C. Cells were incubated with 5 μmol/L di-8-ANEPPS for 10 min at room temperature and protected from light, then re-suspended in fresh Tyrode and used immediately. Confocal XY images were recorded using a 488 nm sapphire laser scanning at 188 lines per second. Emitted fluorescence was directed through a 560 nm long pass filter and collected with a PMT. A rectangular area approximately the length of the cell was selected close to the depth of the nuclei avoiding the surface membrane and a pixel intensity profile was generated using ImageJ. A Fast-Fourier Transform was performed on the intensity profile and a Gaussian best fit curve was fitted to the power spectrum in the frequency domain and used to calculate the amplitude of the first harmonic frequency (Wei *et al.*, 2010). Orientation of tubules was assessed by first subtracting background to enhance contrast, applying median filtering, using automated thresholding to produce a binary image, then skeletonizing images and using the Directionality plugin in ImageJ (Guo & Song, 2014). Tubules oriented 90±5° to the long axis of the cell were considered transverse, while those at 0±5° to the long axis of the cell were considered longitudinal.

Single myocyte electrophysiology

Single RV myocytes were impaled with microelectrodes containing 0.6M KCl (resistance 20-30 MΩ) connected to an Axoclamp 2B amplifier via a HS-2A headstage (Axon Instruments, USA). To assess NCX current myocytes were discontinuous voltage clamped (switching frequency 3 kHz). The SR was primed by depolarising steps of 300 ms duration from -80 mV to +10 mV at 1 Hz. NCX current was then measured as the tail current at -80mV in response to a 20 ms depolarisation to +10 mV. The decay of the tail current was extrapolated to end of the depolarising pulse to exclude the capacitive current (Terrar & White, 1989). NCX current was normalised to cell capacitance. Recordings were made at 36 ± 1ºC.

Biochemical analyses

Right ventricle (RV) free wall samples were snap frozen in liquid N_2 and stored at −80 °C. Samples were homogenized on ice at 30,000 rpm for 4x20 s pulses using a handheld homogenizer (T10, Ika-Werke, Germany) in a buffer containing, in mmol/L: 5 HEPES, 1 EDTA, 5 $MgCl_2$, 0.1% Triton-X100. Protease inhibitors (cOmplete™ Protease Inhibitor Cocktail, Roche, Bazel, Switzerland) and phosphatase inhibitors (Halt™ Phosphatase Inhibitor Cocktail, Thermo Scientific, Waltham, MA) were added to homogenization buffer immediately prior to use. Protein concentration was determined using the Pierce™ BCA Protein Assay Kit (Thermo Scientific, Waltham, MA) in 96 well plates. The optical density of each sample well was recorded at 562 nm in a spectrophotometer (Varioscan Flash, Thermo Scientific, Waltham, MA).

Western blotting

Samples for Western blotting were prepared as previously described (Fowler *et al.*, 2015). Proteins were separated by electrophoresis on SDS-PAGE gel (6-12% acrylamide) in SDS page buffer buffer (0.01 % SDS in 25 mmol/L TRIS, 192 mmol/L glycine) and transferred to a Polyvinylidene difluoride (PVDF) membrane by semi-dry blotting for 85 min at 120 mA per membrane (20% methanol, 0.0375% SDS in 48 mmol/L TRIS, 39 mmol/L glycine) or by wet transfer overnight at 25 V (20% methanol, 25 mmol/L TRIS, 192 mmol/L glycine). Non-specific binding sites were blocked in milk-TRIS buffer (5 % no-fat dried milk powder, 0.1 % Tween-20 in 50 mmol/L TRIS/HCL, 150 mmol/L NaCL) before staining with

specific antibodies. Chemiluminescence was used to detect horseradish peroxidase-linked secondary antibodies before developing on G:Box (Syngene). Band intensities were quantified using Aida Image Analyzer software (Raytest, Germany). Band densities were normalised to the GAPDH loading control and data are presented as % CON values.

Primary antibodies used and their dilution were as follows:
rabbit polyclonal anti-BIN-1 (sc-30099, Santa Cruz Biotechnology, TX) [1:100];
goat polyclonal anti-JP2 (sc-51313, Santa Cruz Biotechnology, TX) [1:500];
rabbit polyclonal anti-GAPDH (G9545, Sigma-Aldrich, UK) [1:100,000]
mouse monoclonal anti-PLN (A010-14, Badrilla, UK) [1.5000]
rabbit polyclonal anti-SERCA2a (A010-20, Badrilla, UK) [1.5000]

Horseradish peroxidase-link secondary antibodies used and their dilutions were as follows:
goat anti-rabbit (Jackson Immuno Research Laboratories Inc., USA) [1:5000];
goat anti-mouse (Jackson Immuno Research Laboratories Inc., USA) [1:10,000]
donkey anti-goat (705-035-147, Stratech) [1:5000]
rabbit anti-goat (A4174, Sigma-Aldrich, UK) [1:2000]

BIN-1 gave a double band associated with amphyphysin II at approximately 100kDa and Bin 1 splice variant at approximately 75kDa. The densities of these 2 bands were added and normalized to GAPDH. The double band for phospholamban in Fig 4 corresponds to the pentameric and monomeric forms. All samples used for analysis of a particular target were loaded on the same gel. Blots in Fig 4 were stripped and re-probed for GAPDH. The blot in Fig 5D was stripped and re-probed for JP2 and was normalized to the original GAPDH signal in Fig 5D.

Measurement of mRNA using Real-Time PCR

Total RNA extraction was performed following the Qiagen mini-kit protocol for striated muscle, with the exception that an additional volume of RLT buffer was added to the proteinase K solution before binding of the RNA onto the mini-columns. Integrity, quantity and purity of the isolated RNA was assessed using NanoDrop microspectrophotometry. cDNA was prepared from 7.5 µg of total RNA with random priming using Superscript III first-strand synthesis system (Invitrogen, Life Technologies, Rockville, MD) and diluted 1:2 in TE (10 mmol/L Tris·HCl pH 7.5 and 1 mmol/L EDTA) before use in real-time PCR.

Real-time RT-PCR was performed using 10 Real Time Ready Custom Panels 384 (Roche). Each card consisted of 384 wells, preloaded with predesigned fluorogenic probes and primers, configured to allow detection of 96 transcripts for three experimental and one calibrator sample. Our calibrator sample consisted of a mixture of cDNA from RV regions of 2 CON, MCT+BB and MCT hearts. Each well of the 384 custom panel was loaded with 10 µl of a 1:1 mixture of cDNA (equivalent to 40 ng input RNA) and LightCycler 480 Probes Master Mix (Roche) using the EpMotion 5070 Liquid Handling Workstation (Eppendorf). PCR was performed according to the recommended protocol (pre-incubation at 95 °C for 10 min, amplification for 45 cycles at 95 °C for 10 s, 60 °C for 30 s and 72 °C for 1 s followed by a cooling step at 40 °C for 30 s) on the LightCycler 480ii Instrument (Roche). Data were collected with the LightCycler 480 software and analyzed using the basic relative quantification module based on the ΔΔCT-Method. Transcript expression was normalized to the housekeeping genes 18S and GAPDH in the test samples and then made relative to the normalized signal level in the corresponding calibrator sample.

Statistics

Required sample sizes were calculated *a priori* using preliminary data or published results from the scientific literature. We tested for clustering of cell data by animal using the intra-class correlation (ICC) method (Sikkel *et al.*, 2017). We found a low ICC for salient results (e.g. ICC <5% for myocyte width, <5% for t-tubule density, 15% for Ca^{2+} spark frequency) indicating minimal clustering of data by animal, therefore statistical analyses were performed at the level of the myocyte or animal.

Number of rats or hearts used in each experiment are provided in the figure legends; in isolated cell experiments the number of cells used for statistical analysis is presented inset in the figure. Statistical analysis was performed with n = myocyte or animal number. Statistical analyses were performed in SigmaStat 3.5 (Systat Software, San Jose, CA) and Prism 6 (Graphpad, La Jolla, CA). Tests for normal distribution were performed and parametric statistical tests were performed where data followed a normal distribution, otherwise data was $\log_{10}$ transformed and normality was re-tested. Parametric statistical tests were used on transformed data which passed tests for normality. In cases where data still did not pass tests for normality, equivalent non-parametric statistical tests were used on the original data. Curves of survival to heart failure signs were compared using a Mantel-Cox test. T-tests or one-way ANOVA, or non-parametric equivalents, were used to compare two or more groups. Categorical events were compared using Fisher's Exact test. Two-way repeated measures ANOVA were used to compare sequential measures on the same cells. *Post-hoc* multiple comparison tests recommended by the statistical packages were used. Following parametric ANOVA, the Holm-Sidak multiple comparison test was used. Following non-parametric ANOVA either Tukey's (group sizes equal) or Dunn's (group sizes unequal) correction test was used. $P<0.05$ was considered significant. Data are presented as mean±SEM.

Supplementary Results

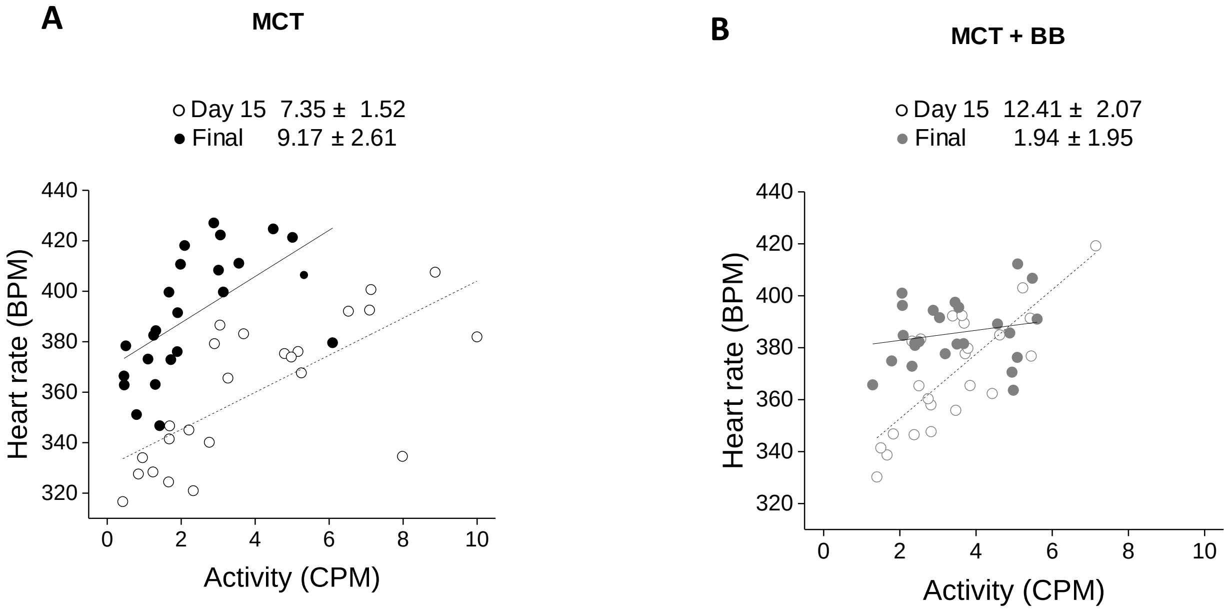

Fig S1. BB flattens the heart rate-activity relationship in freely moving rats. Mean heart rate and activity (in activity counts per minute) were recorded each hour over a 24h period on day 15 prior to vehicle (MCT) or BB treatment (MCT+BB) and on the final experimental day. **A** In MCT rats, there was a significant positive correlation between heart rate and activity at both the start and end of vehicle treatment ($P<0.01$). **B** In MCT+BB rats prior to the first dose of BB, heart rate was positively correlated with activity ($P<0.001$). However, at the end of treatment with BB, an increase in activity did not cause a corresponding increase in heart rate ($P=0.33$). N= 3 MCT and 8 MCT+BB animals. Slopes of the relationships are given in the figure.

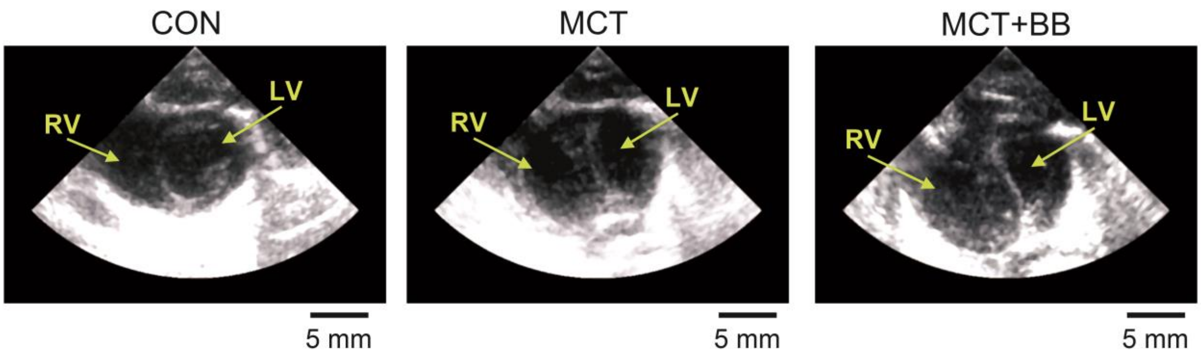

Fig S2. Echocardiography images recorded in the parasternal short axis view. PAH resulted in increased diameter of RV chamber and a hypocontractile RV wall with increased thickness.

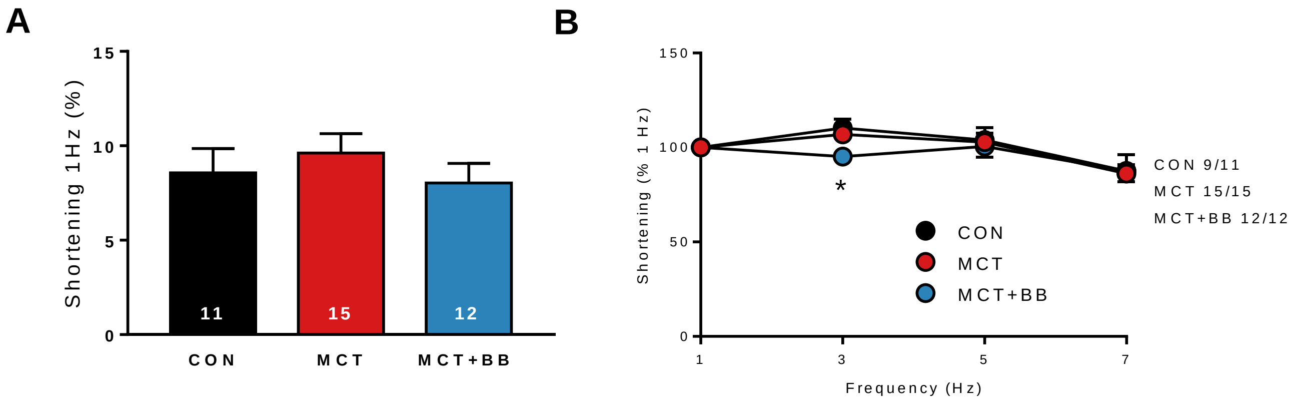

Fig S3. Contractility of left ventricular myocytes was not affected by MCT. Sarcomere shortening was recorded in left ventricular (LV) myocytes from CON, MCT and MCT+BB rats. **A** At 1 Hz there was no difference in sarcomere shortening of LV myocytes. **B** The response to faster pacing was broadly similar in each group. The number of myocytes contracting regularly at 7 Hz is shown. N = 3 CON, 4 MCT, 5 MCT+BB rats. Two way RM ANOVA, * P<0.05 vs CON.

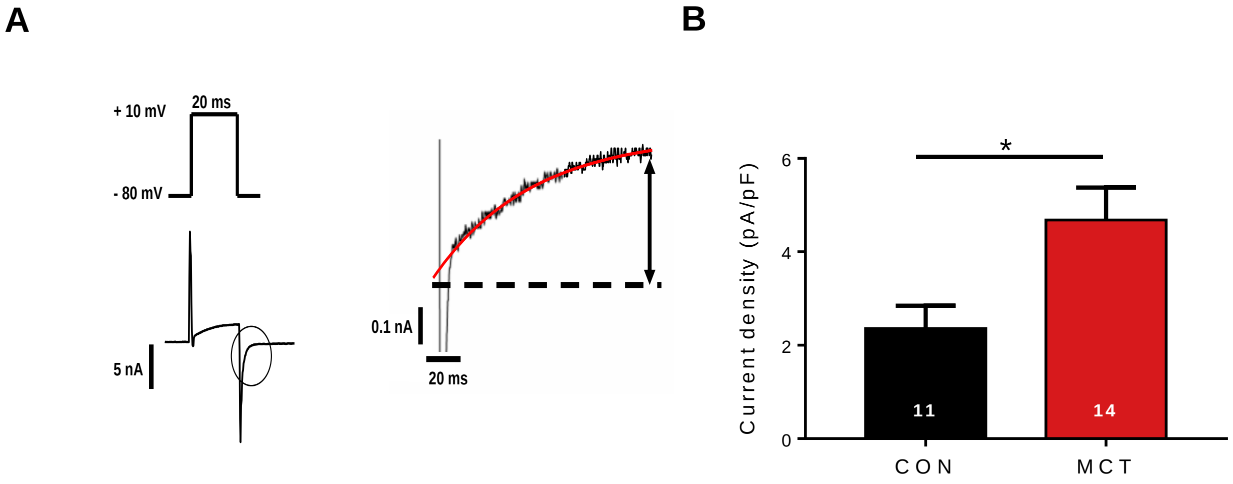

Fig S4. Increased NCX current in MCT myocytes. **A** Representative recording showing NCX current measured after a 20 ms depolarising step to +10 mV from a holding potential of -80 mV following 300ms priming pulses. NCX amplitude is marked by the double headed arrow **B** NCX current was greater in MCT cells than CON following the same priming protocol at 1Hz (N= 11 CON and 14 MCT myocytes). Unpaired t-test * P<0.05.

	CON day 15	MCT day 15
Rats/group	9	12
Heart rate (bpm)	408 ± 7	403 ± 8
PAAT (ms)	35.7 ± 2.7	28.7 ± 1.1*
Systolic pressure (mmHg)	52.0 ± 4.9	64.8 ± 2.1*
Stroke volume (μL)	129 ± 14	117 ± 8
Cardiac output (mL/min)	53.0 ± 6.1	46.9 ± 3.1
RV thickness diastole (mm)	0.68 ± 0.04	0.80 ± 0.05#
RVID diastole (mm)	2.13 ± 0.24	3.00 ± 0.43
RV area diastole (cm^2)	0.08 ± 0.01	0.13 ± 0.02*

Table S1 RV echocardiography prior to BB treatment showed established PAH and RV hypertrophy in MCT treated rats. Echocardiography was used to non-invasively verify the presence of PAH at the start of BB treatment. Pulmonary artery acceleration time (PAAT) was reduced, equating to an estimated increase of systolic pressure of 12.8 mmHg in MCT compared to CON. RV diastolic area was also increased, indicative of early RV remodelling in response to PAH. Unpaired t-test #P=0.09, *P<0.05.

	CON	MCT	MCT+BB
Rats/group	26	21	26
Body weight (g)	321 ± 6	274 ± 5*	270 ± 5*
Weight loss (g)	—	10 ± 1.4	4.9 ± 1.4†
Heart weight (g)	1.26 ± 0.03	1.47 ± 0.05*	1.45 ± 0.04*
Lung weight (g)	2.45 ± 0.19	2.78 ± 0.10	2.76 ± 0.11
RV (g)	0.22 ± 0.01	0.35 ± 0.03*	0.30 ± 0.02*
LV+S (g)	0.85 ± 0.04	0.83 ± 0.04	0.78 ± 0.03
Heart/body weight (mg/g)	3.92 ± 0.06	5.37 ± 0.16*	5.38 ± 0.17*
Lung/body weight (mg/g)	7.60 ± 0.53	10.10 ± 0.23*	10.23 ± 0.37*
RV/body weight (mg/g)	0.70 ± 0.02	1.27 ± 0.07*	1.13 ± 0.07*
LV+S/body weight (mg/g)	2.64 ± 0.09	3.00 ± 0.09*	2.90 ± 0.08
RV:LV+S ratio	0.27 ± 0.01	0.43 ± 0.03*	0.39 ± 0.02*

Table S2. Animal and organ characteristics of rats on the final experimental day. There was less weight loss, from highest weight recorded in each animal, in MCT+BB rats compared to MCT by the final experimental day. RV weights and RV:LV+septal ratios were less in MCT+BB than MCT but these differences were not statistically significant. One-way ANOVA. *P<0.05 vs CON, †P<0.05 vs MCT.

	CON	MCT	MCT+BB
Rats/group	9	7	5
Heart rate (bpm)	409 ± 9	364 ± 12*	340 ± 9*
PA acceleration time (ms)	35.9 ± 3.0	20.5 ± 1.5*	26.5 ± 1.8*
Systolic pressure (mmHg)	51.5 ± 5.5	79.6 ± 2.8*	68.7 ± 3.4*
Stroke volume (µL)	140 ± 13	52 ± 9*	100 ± 13*†
Cardiac output (mL/min)	57.7 ± 6.4	18.9 ± 3.4*	34 ± 4.4*#
RV thickness diastole (mm)	0.72 ± 0.04	1.43 ± 0.07*	1.08 ± 0.06*†
RVID diastole (mm)	2.47 ± 0.26	5.00 ± 0.26*	4.68 ± 0.19*
RV area diastole (cm^2)	0.11 ± 0.01	0.34 ± 0.04*	0.30 ± 0.04*

Table S3. RV echocardiography on the final experimental day revealed reduced RV hypertrophy and improvements in function following BB treatment. Echocardiography was performed on day 23 ± 1 (CON and MCT+BB) or on the day of heart failure signs (MCT). Systolic pressure was estimated from pulmonary artery (PA) acceleration time. Stroke volume and cardiac output were estimated from PA Doppler flow measurements. RVID, right ventricle inner diameter. One way ANOVA *P<0.05vs CON, †P<0.05 vs MCT, #P=0.09.

	CON	MCT	MCT+BB
Number of rats	6	9	9
RV myocytes			
Number of cells	79	70	73
Length (µm)	117.3 ± 1.3	108.8 ± 1.4***	114.2 ± 1.4†
Width (µm)	22.5 ± 0.4	27.5 ± 0.5***	24.5 ± 0.4**†††
Volume (pL)	22.9 ± 0.9	31.3 ± 1.1***	26.3 ± 1.1*††
Sarcomere length (µm)[1]	1.92 ± 0.01	1.79 ± 0.01***	1.87 ± 0.01**†††
LV myocytes			
Number of cells	64	66	64
Length (µm)	117.1 ± 1.5	114.6 ± 1.5	117.7 ± 1.5
Width (µm)	23.5 ± 0.5	23.5 ± 0.4	23.1 ± 0.5
Volume (pL)	24.8 ± 1.0	24.3 ± 1.0	24.4 ± 1.2
Sarcomere length (µm)[1]	1.89 ± 0.01	1.86 ± 0.01	1.90 ± 0.01††

Table S4. Geometry of isolated myocytes. RV and LV myocytes were isolated separately and their dimensions measured. Volume was calculated from myocyte length and width as described in text. RV myocyte width and volume were greater in MCT than CON, but were significantly reduced following BB treatment. There were no differences in LV myocyte geometry between groups. Resting sarcomere length in RV cells was shorter in MCT than CON, but was partially restored in MCT+BB. [1]Sarcomere length was measured in n = 30 cells from each group. One-way ANOVA, * P<0.05, ** P<0.01, *** P<0.001 vs CON, † P<0.05, †† P<0.01, ††† P<0.001 vs MCT.

Reference List

Caldwell JL, Smith CE, Taylor RF, Kitmitto A, Eisner DA, Dibb KM, & Trafford AW (2014). Dependence of cardiac transverse tubules on the BAR domain protein amphiphysin II (BIN-1). *Circ Res* **115**, 986-996.

Cannell MB, Cheng H, & Lederer WJ (1994). Spatial non-uniformities in [Ca2+]i during excitation-contraction coupling in cardiac myocytes. *Biophys J* **67**, 1942-1956.

Feigenbaum H (2004). *Feigenbaum's Echocardiography* Lippincott Williams & Wilkins.

Fowler ED, Benoist D, Drinkhill MJ, Stones R, Helmes M, Wust RC, Stienen GJ, Steele DS, & White E (2015). Decreased creatine kinase is linked to diastolic dysfunction in rats with right heart failure induced by pulmonary artery hypertension. *J Mol Cell Cardiol* **86**, 1-8.

Gomez-Arroyo JG, Farkas L, Alhussaini AA, Farkas D, Kraskauskas D, Voelkel NF, & Bogaard HJ (2012). The monocrotaline model of pulmonary hypertension in perspective. *Am J Physiol Lung Cell Mol Physiol* **302**, L363-L369.

Guo A & Song LS (2014). AutoTT: automated detection and analysis of T-tubule architecture in cardiomyocytes. *Biophys J* **106**, 2729-2736.

Hardziyenka M, Campian ME, de Bruin-Bon HA, Michel MC, & Tan HL (2006). Sequence of echocardiographic changes during development of right ventricular failure in rat. *J Am Soc Echocardiogr* **19**, 1272-1279.

Jones JE, Mendes L, Rudd MA, Russo G, Loscalzo J, & Zhang YY (2002). Serial noninvasive assessment of progressive pulmonary hypertension in a rat model. *Am J Physiol Heart Circ Physiol* **283**, H364-H371.

Kong CH, Soeller C, & Cannell MB (2008). Increasing sensitivity of Ca2+ spark detection in noisy images by application of a matched-filter object detection algorithm. *Biophys J* **95**, 6016-6024.

Maarman G, Lecour S, Butrous G, Thienemann F, & Sliwa K (2013). A comprehensive review: the evolution of animal models in pulmonary hypertension research; are we there yet? *Pulm Circ* **3**, 739-756.

Natali AJ, Wilson LA, Peckham M, Turner DL, Harrison SM, & White E (2002). Different regional effects of voluntary exercise on the mechanical and electrical properties of rat ventricular myocytes. *J Physiol* **541**, 863-875.

Nogueira-Ferreira R, Vitorino R, Ferreira R, & Henriques-Coelho T (2015). Exploring the monocrotaline animal model for the study of pulmonary arterial hypertension: A network approach. *Pulm Pharmacol Ther* **35**, 8-16.

Reagan-Shaw S, Nihal M, & Ahmad N (2008). Dose translation from animal to human studies revisited. *FASEB J* **22**, 659-661.

Rudski LG, Lai WW, Afilalo J, Hua L, Handschumacher MD, Chandrasekaran K, Solomon SD, Louie EK, & Schiller NB (2010). Guidelines for the echocardiographic assessment of the right heart in adults: a report from the American Society of Echocardiography endorsed by the European Association of Echocardiography, a registered branch of the European Society of Cardiology, and the Canadian Society of Echocardiography. *J Am Soc Echocardiogr* **23**, 685-713.

Ryan JJ, Marsboom G, & Archer SL (2013). Rodent models of group 1 pulmonary hypertension. *Handb Exp Pharmacol* **218**, 105-149.

Satoh H, Delbridge LM, Blatter LA, & Bers DM (1996). Surface:volume relationship in cardiac myocytes studied with confocal microscopy and membrane capacitance measurements: species-dependence and developmental effects. *Biophys J* **70**, 1494-1504.

Sikkel MB, Francis DP, Howard J, Gordon F, Rowlands C, Peters NS, Lyon AR, Harding SE, & MacLeod KT (2017). Hierarchical statistical techniques are necessary to draw reliable conclusions from analysis of isolated cardiomyocyte studies. *Cardiovasc Res* **113**, 1743-1752.

Terrar DA & White E (1989). Changes in cytosolic calcium monitored by inward currents during action potentials in guinea-pig ventricular cells. *Proc R Soc Lond B Biol Sci* **238**, 171-188.

Varro A, Negretti N, Hester SB, & Eisner DA (1993). An estimate of the calcium content of the sarcoplasmic reticulum in rat ventricular myocytes. *Pflugers Arch* **423**, 158-160.

Wei S, Guo A, Chen B, Kutschke W, Xie YP, Zimmerman K, Weiss RM, Anderson ME, Cheng H, & Song LS (2010). T-tubule remodeling during transition from hypertrophy to heart failure. *Circ Res* **107**, 520-531.

Wier WG, Cannell MB, Berlin JR, Marban E, & Lederer WJ (1987). Cellular and subcellular heterogeneity of [Ca2+]i in single heart cells revealed by fura-2. *Science* **235**, 325-328.
